# Association between alcoholic interventions and abstinence rates for alcohol use disorders: A meta-analysis

**DOI:** 10.1097/MD.0000000000013566

**Published:** 2018-12-14

**Authors:** Jiamin Gao, Jun Cao, Tao Guo, Yunyue Xiao

**Affiliations:** a Department of Emergency, Huashan Hospital, Fudan University, Shanghai; b Department of Hepatobiliary and Pancreatic Surgery, Department of General Surgery, Zhongnan Hospital of Wuhan University, Wuhan; c Department of Clinical Psychology, Southwest Hospital, Third Military Medical University (Army Medical University), Chongqing, China.

**Keywords:** abstinence rates, alcoholic interventions, network meta-analysis

## Abstract

**Objective:**

The aim of present study is to quantitatively evaluate the association between different interventions and abstinence rates based on network meta-analysis.

**Method:**

Following PRISMA guidelines, randomized clinical trials that compared different alcoholic interventions for alcohol use disorders associated with abstinence rates in treatment sessions or/and follow-up sessions were recruited. Main data synthesis was performed by Bayesian random-effects network meta-analyses, and the relative ranking of interventions was estimated by cumulative probability *P* values (SUCRA). Funnel plot symmetry was used to detect publication bias. Moreover, pair-wised comparison was also conducted to determine the statistical difference and forest plots were generated to calculate the differences between the groups. The Grades of Recommendations Assessment, Development and Evaluation (GRADE) criteria were utilized for the recommendations of evidence from pairwise direct comparisons.

**Results:**

A total of 137 RCTs containing 27,282 participants and 8 variations of psychotherapy, pharmacotherapy, contingency management, and brief intervention used as treatment interventions were included. In summary, contingency management plus psychotherapy was demonstrated to be effective and possessed the best rank of achieving the highest abstinence rate in treatment sessions (SUCRA, 0.61). Pharmacotherapy plus psychotherapy also revealed its efficacy and was associated with the highest abstinence rate in follow-up sessions (SUCRA, 0.40). More importantly, psychotherapy alone was demonstrated not to be associated with higher abstinence rates in both treatment (OR, 1.052; 95% CI, 0.907–1.220) and follow-up sessions (OR, 0.967; 95% CI, 0.552–1.693), yet pharmacotherapy seemed to be the only intervention associated with higher abstinence rates compared to controls in both sessions (treatment session: OR, 1.074; 95% CI, 1.002–1.152) (follow-up session: OR, 1.442; 95% CI, 1.094–1.900).

**Conclusions:**

Contingency management plus psychotherapy and pharmacotherapy plus psychotherapy were demonstrated to be associated with the highest abstinence rates in treatment sessions and follow-up sessions, respectively. However, contingency management and pharmacotherapy seemed to be the substantial crucial factors allowing for the maintenance of the highest abstinence rates in respective sessions, although we need more evidence for further validation.

## Abstract 2

Supplemental Digital Content is available in the text

## 1 Introduction

Alcohol use disorders (AUDs), encompassing various serious forms of consumption, is the leading preventable cause of morbidity and a major contributor to health care costs,^[1–3]^ but most individuals with an AUD never receive treatment.^[4]^ AUDs remain widespread in developed countries, and they are the key factor in liver cirrhosis.^[5]^ Alcohol affects the health of not only the drinking individual but also the fetus in pregnant women. The neurotoxic effects of alcohol may cause a range of congenital defects, including fetal alcohol spectrum disorders and fetal death, stillbirth, and infant and child mortality.^[6]^The AUD is also a psychiatric diagnosis^[7–8]^ which is similar to other chronic illnesses, AUDs have physiological and behavioral components as well as relapse rates.^[9]^ Therefore, AUDs may cause a wide range of medical, psychological, social, personal, and economic problems.

Currently, various alcoholic interventions are reportedly aiming to reduce alcohol abuse. For example, psychotherapy is described as psychologically based interventions that exclude any pharmacological treatments and are aimed at reducing consumption behavior or alcohol-related problems.^[10–12]^ It comprises systematic and stepped continuous therapy but requires costly support from experienced psychologists. Pharmacotherapy was also considered physiologically effective, and some medications were approved by the US Food and Drug Administration (FDA).^[13–14]^ Additionally, some brief interventions were applied in the community population, such as simple group counseling, brief mobile electronic program monitoring, and even brief educational interventions derived from psychotherapy.^[15–16]^ All these approaches are now utilized for treating AUDs, but at the same time, the effectiveness of these interventions compared with one another is controversial. More importantly, the association of different alcoholic interventions and abstinence rates has not been fully analyzed.

Since the emergence of the concept of AUDs, hundreds of randomized controlled trials (RCTs) of various alcoholic interventions were reported. However, the availability of alcoholic management options poses a challenge when making evidence-based management decisions due to superior interventions not yet having been determined, although there was an initiative proposed years ago.^[17]^ Therefore, a comprehensive systematic review and Bayesian network meta-analysis were conducted to summarize the evidence from RCTs comparing various alcoholic interventions for the elucidation of the association between alcoholic abstinence rates and interventions.

## 2 Methods

This study was performed according to the Preferred Reporting Items for Systematic Reviews and Meta-analyses (PRISMA) statement extension for network meta-analysis.^[18]^ Moreover, this review was registered online at the Research Registry Center with obtained UIN number review registry 532.

### 2.1 Study eligibility criteria

Randomized clinical trials (RCTs) that compared any alcoholic interventions with no treatment control or with each other were considered eligible if they reported ≥ 2 weeks of treatment or/and follow-up sessions.

Non-RCTs or observational trials, trials without sufficient parametric data, and trials without interesting (e.g., preclinical pharmacotherapy testing for hours; studies focusing on holidays, anniversaries, or ceremonies) were excluded. In addition, papers of reviews, comments and basic science were also excluded.

### 2.2 Selection of studies

Three globally recognized databases (MEDLINE, Embase, Cochrane Central) were searched from inception to December 2017 without publication status restriction. The search strings were based on MeSH terms (example search strategy in MEDLINE is presented in Table S1 of supporting information). Full English texts had to be addressed if the trial was considered for inclusion. Study retrieval and identification were conducted by all members of our group under the guidance of the same eligibility criteria and search strategy.

### 2.3 Data extraction and outcomes of interest

General information and intervention-related characteristics were abstracted into a standardized form (Table S2 in the supporting information). For the outcome of interest, we preferred abstinence rates rather than other subjective data after unanimous discussion. We deemed abstinence the terminal purpose of alcoholic treatment, and the measurement of abstinence rates may be more accurate than continuous variables (such as the amount of drinking). Furthermore, even some objective parametric data (such as drinking frequency, percent of drinking days) were considered inhomogeneous in different trials (because of the different definitions of minimal drinking); thus, these data were not appropriate for data synthesis. Nevertheless, data on abstinence rates, which was defined as no alcohol use, were essentially homogeneous for pooled estimation.

For extraction of outcomes, the raw data of abstinence rates in treatment sessions and in follow-up sessions were individually extracted for separate comparisons. Treatment sessions were defined as the whole therapeutic period under respective alcoholic intervention until the end of the treatment. Follow-up sessions were considered from the endpoint of treatment to the end of follow-up without any interventions in each group. Self-reports of no alcohol use, continuous negative detection of monitoring devices, or/and rates of continuous negative bio-sample testing were considered available abstinence data for comparison. Data of intention-to-treat patients were preferred for extraction. In addition, for those papers presenting only survival curves, Engauge Digitizer (version 4.1) was used to extract raw data of abstinence proportions.^[19–20]^ Data extraction was performed by 2 independent investigators, and any debates were resolved by group discussion.

### 2.4 Quality assessment and recommendation of evidence

Included trials were assessed by the Cochrane Risk of Bias assessment tool^[21]^ to address the bias risk of individual studies with following requirements:

1. Free of selection bias,
2. Free of performance bias,
3. Free of detection bias,
4. Free of attrition bias,
5. Free of reporting bias, and
6. Free of other bias.

A graphic summary of the overall and study-level risk of bias was conducted using Review Manager Software (version 5.3).

To confirm the reliability and quality of the present study, the Grades of Recommendations Assessment, Development and Evaluation (GRADE) criteria were selected to assess the methodological quality of evidence.^[22]^ Five factors that may reduce the quality of evidence were considered (research limitations, inconsistent findings, uncertain direct evidence, inaccuracy or wide confidence intervals, and publication bias). Additionally, 3 factors that can enhance the quality of evidence were reviewed (effect size, possible confounding factors, and dose-effect relationship). Furthermore, in this approach, the rating of indirect estimate starts at the lowest rating of the 2 pairwise estimates that contribute as 1st-order loops to the indirect estimate but can be downgraded further for imprecision.^[23]^ All investigators assessed the quality of the examined studies through discussion until reaching agreement. Explanations for the Cochrane Summary of Findings Table of the GRADE system were made by the software GRADE profiler (version 3.6).

### 2.5 Statistical analysis

Indirect pooled estimation of alcoholic interventions was conducted to make comprehensive network comparisons based on the Bayesian theorem. This approach can be considered an extension of the traditional pairwise meta-analysis because it incorporates both direct and indirect information through a common comparator to obtain estimates of the relative interventional effects on multiple intervention comparisons.^[24–25]^ The surface under the cumulative ranking (SUCRA) probabilities of the *P* values were presented to clarify the pros and cons of different alcoholic interventions. The highest *P* value represented the possibility of achieving the highest abstinence rate, and these methods were described previously.^[26–27]^ Odds ratios (ORs) derived from network meta-analysis were calculated to exhibit the comparison of different interventions. Publication bias was assessed by examining funnel-plot symmetry. Inconsistency model approach was used to test the consistency of main results based on the node-splitting analysis. No statistical inconsistency was shown at *P* > .05.^[28]^

Moreover, sensitivity analyses were performed to assess the robustness of the main outcomes. Random effects Bayesian network meta-analyses were also performed among the following: only USA trials; treatment and follow-up sessions ≥ 12 weeks; only continuous abstinence rates. All relative ORs and credible intervals (CIs) were estimated for sensitivity analysis.

Direct meta-analysis was conducted to complete direct comparisons for further investigation. In this condition, heterogeneity (*I*^2^ index statistic) in the study design was used to estimate a data mode for using fixed- (*I*^2^ < 50%) or random- (*I*^2^ > 50%) effects models.^[29]^ The associated 95% CIs were calculated, the level of statistical significance was set at *P* < .05, and all statistical tests were 2-sided.

**Figure 1:**
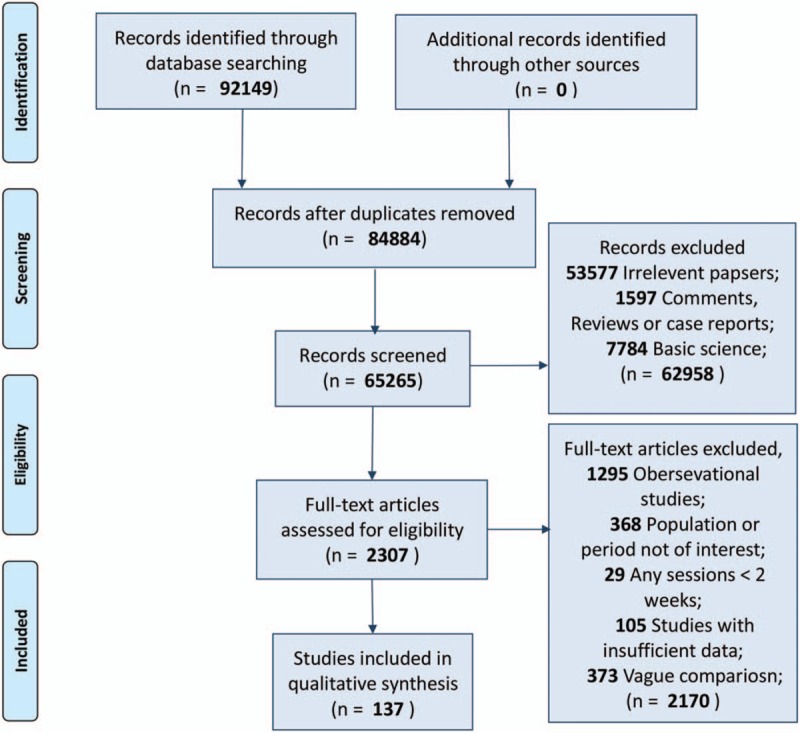
Literature search and study selection flow diagram.

Data manipulation and statistical analyses of network meta-analysis and pairwise analysis were conducted using the Stata software package (version 12.0).^[30]^ The data model was verified by using the automated software Aggregate Data Drug Information System (ADDIS, version 1.16) (Fig. 1).

## 3 Results

**Figure 2:**
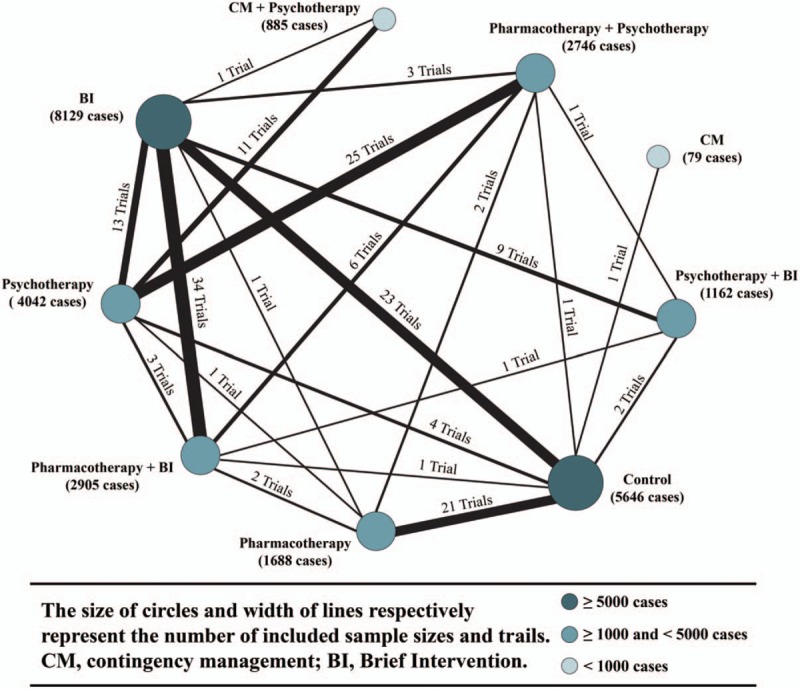
Network plots of included trials with available direct comparisons.

The searches identified 92,149 records, of which 2307 were considered relevant clinical studies after titles and abstracts were reviewed. Eventually, based on the review of full texts, 137 trials containing 27,282 participants were included for quantitative analysis. Most of them (127 trials) were 2-arm studies, and the rest of them were 3- (5 trials) or 4-arm (5 trials) studies. Moreover, given the intensive review of studies, all relative interventions were classified into 5 categories: psychotherapy (including any systematic series of psychotherapy sessions, i.e., full cognitive behavioral therapy or/and motivational enhancement therapy, motivational interviewing, and 12-step facilitation session); pharmacotherapy (any pharmacological application for alcohol abstinence, such as baclofen, naltrexone, nalmefene, disulfiram, and acamprosate); CM (contingency management, containing any positive incentives with prizes or money aiming at abstinence); BI (brief intervention, which indicates non-systematic or simple session intervention, e.g., continuous or intermittent brief advice or education, brief session deprived of psychological intervention, nursing supervision, web or telephone-based brief self-monitoring program, community or family visiting, group or face-to-face simple supportive counseling, periodic social interaction, and sports encouragement such as yoga and aerobic exercise); and control (no treatment for negative comparison, e.g., placebo application, minimal advice or education at recruitment, assessment only). We categorized studies according to these 5 interventions and summed 4 additional combinations (pharmacotherapy plus BI, psychotherapy plus BI, pharmacotherapy plus psychotherapy, and CM plus psychotherapy) for the final estimation. The available direct comparisons and network plot of included trials are shown in Figure 2.

### 3.1 Study characteristics and quality assessment

In general, all included 137 RCTs were published from 1979 to 2017, containing 27,282 cases. For 8 included interventions and controls, the sample sizes ranged between 79 and 8129 (Fig. 2). Studies were conducted in a wide range of countries but were mostly reported from developed countries. A total of 130 studies provided available data for treatment sessions, and 35 reported follow-up sessions (28 studies reported both). The details were presented in the supporting information (Table S2 in supporting information).

For quality assessment, 82 trials (60%) were conducted with random sequence generation, but only 57 trials (42%) applied blinding methods. Overall, included studies were considered to be at high risk of bias. The details of the overall and study-level risk of bias assessments were summarized (Figure S1 in supporting information).

### 3.2 The interventions achieving the highest alcoholic abstinence rates

**Figure 3:**
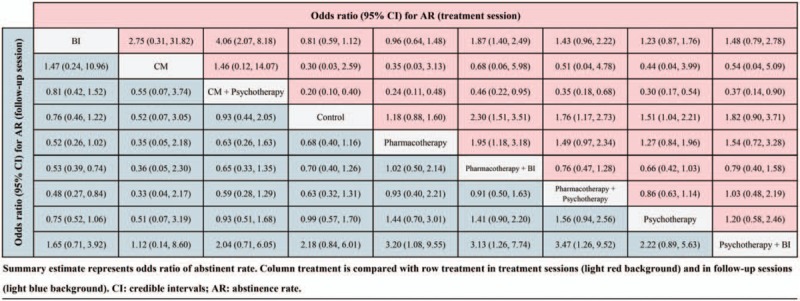
Network comparison of various alcoholic interventions with abstinence rate in treatment sessions and follow-up sessions.

**Figure 4:**
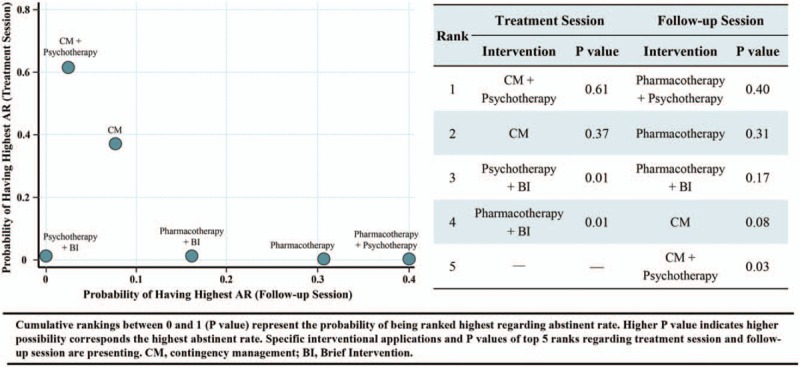
Cumulative *P* values and best rank of respective interventions achieving alcoholic abstinence.

For the abstinence rates in treatment sessions, 130 trials containing 26,097 cases reported relevant parametric data. Head-to-head comparisons between the different therapeutic options were depicted as network plots (Figure S2A in supporting information). Network odds ratios (ORs) for each possible comparison of all 8 interventions and the control were estimated and presented in Figure 3. The results indicated that, compared with the control, the other 8 included interventions were associated with a higher odds of abstinence. In addition, compared to the control, psychotherapy plus BI was associated with an OR of 1.82 (95% credible interval [CI], 0.90–3.71), psychotherapy with an OR of 1.51 (95% credible interval [CI], 1.04–2.21), pharmacotherapy plus psychotherapy with an OR of 1.76 (95% credible interval [CI], 1.17–2.73), pharmacotherapy plus BI with an OR of 2.30 (95% credible interval [CI], 1.51–3.51), and pharmacotherapy with an OR of 1.18 (95% credible interval [CI], 0.88–1.60). In addition, the control was associated with lower odds than CM plus psychotherapy (OR, 0.20 [95% CI, 0.10–0.40]), CM (OR, 0.030 [95% CI, 0.03–2.59]), and BI (OR, 0.81 [95% CI, 0.59–1.12]) (Figure 3). Meanwhile, based on cumulative *P* values, CM plus psychotherapy was ranked 1st in its association with the highest alcohol abstinence rate in treatment sessions (cumulative *P* = .61), followed by CM (*P* = .37), psychotherapy plus BI (*P* = .01), and pharmacotherapy plus BI (*P* = .01) (Fig. 4).

On the other hand, 35 trials with 6329 participants reported abstinence rates in follow-up sessions. The network plot revealed the available direct comparison (exhibited in Figure S2B in supporting information). After quantitative comparison, the network meta-analysis suggested that the control was associated with lower odds of abstinence than 4 interventions: pharmacotherapy, with an OR of 0.68 (95% CI, 0.40–1.16); pharmacotherapy plus BI, with an OR of 0.70 (95% CI, 0.40–1.26); pharmacotherapy plus psychotherapy, with an OR of 0.63 (95% CI, 0.32–1.31); and psychotherapy, with an OR of 0.99 (95% CI, 0.57–1.70) (Fig. 3). Moreover, the results of network meta-analysis revealed that pharmacotherapy plus psychotherapy was associated with the highest probability of achieving the highest abstinence rates in follow-up sessions (with cumulative *P* = .40), followed by pharmacotherapy (*P* = .31), pharmacotherapy plus BI (*P* = .17), CM (*P* = .08) and CM plus psychotherapy (*P* = .03) (Fig. 4).

### 3.3 Sensitivity analysis

To ensure the reliability of the main results, we conducted sensitivity analyses based on the following issues: USA trials (because more than half of the trials came from the United States); sessions ≥ 12 weeks (since most of the treatment or/and follow-up periods were over 12 weeks, and some interventions may reveal longer-term benefits, e.g., psychotherapy); and continuous abstinence (most included trials reported these data, and it may provide a better representation of abstinence). The relative ORs were estimated and presented in detail (Table S3 in supporting information). Overall, the results of sensitivity analysis were similar to the main outcomes.

### 3.4 Direct meta-analysis for the validation of the main results

To further validate our main results, we conducted a direct meta-analysis to verify the significant differences. We 1st estimated 60 reports from 43 trials (Figure S3A and Table S4 in supporting information), which covered 6 interventions directly compared with controls, and the results indicated that the application of the intervention could significantly increase the alcoholic abstinence rate with significant differences in treatment sessions (OR, 1.260 [95% CI, 1.091–1.455]) with high heterogenicity (*I*^2^ = 67.3%) (Table S4 in supporting information). Moreover, 16 reports from 12 trials covering 5 alcohol interventions compared with controls in follow-up session were pooled-analyzed (Figure S3B and Table S4 in supporting information). Overall, the application of alcohol interventions could significantly enhance abstinence rates compared with controls (OR, 1.252 [95% CI, 1.012–1.550]) with low heterogenicity (*I*^2^ = 12.2%) (Table S4 in supporting information) in follow-up sessions. Next, we further separated each intervention to make a comparison with controls in the treatment session. The results suggested that pharmacotherapy plus psychotherapy (OR, 1.178 [95% CI, 1.002–1.386]), pharmacotherapy (OR, 1.074 [95% CI, 1.002–1.152]), CM (OR, 1.306 [95% CI, 1.048–1.627]), BI (OR, 1.064 [95% CI, 1.003–1.128]), and psychotherapy plus BI (OR, 1.500 [95% CI, 1.055–2.133]) revealed significant differences for abstinence rates compared to controls in treatment sessions, yet psychotherapy did not (OR, 1.052 [95% CI, 0.907–1.220]). Meanwhile, we also observed that the application of pharmacotherapy exhibited significant differences with regard to abstinence rates in follow-up sessions (OR, 1.442 [95% CI, 1.094–1.900]), yet BI (OR, 1.062 [95% CI, 0.928–1.216]), rather than the application of psychotherapy (OR, 0.967 [95% CI, 0.552–1.693]) (Table S4 in supporting information). Therefore, we may understand that the application of some certain interventions may not reveal any benefits in both treatment and follow-up sessions.

As mentioned above, we addressed the finding that CM plus psychotherapy and pharmacotherapy plus psychotherapy were associated with the highest alcohol abstinence rates in the treatment session and follow-up session, respectively. To statistically determine their validation of practical benefit, we performed direct comparisons between CM plus psychotherapy and other interventions in treatment sessions and comparisons between pharmacotherapy plus psychotherapy and other interventions in follow-up sessions. A total of 11 reports from 8 trials reported the findings of CM plus psychotherapy versus other interventions (Figure S4A in supporting information). Pooled estimation demonstrated CM plus psychotherapy to have significant benefits in enhancing alcohol abstinence rates compared with other interventions in the treatment session (OR, 2.191 [95% CI, 1.290–3.720]) with high heterogenicity (*I*^2^ = 70.7%). On the other hand, based on 9 reports from 6 trials, a direct meta-analysis of pharmacotherapy plus psychotherapy versus other interventions in follow-up sessions was conducted (Figure S4B in supporting information). The results illustrated that pharmacotherapy plus psychotherapy showed significant efficacy in increasing alcohol abstinence rates compared to other interventions (OR, 1.409 [95% CI, 1.079–1.840]) with low heterogenicity (*I*^2^ = 0%) (Table S5 in supporting information). For further exploration, we next separately compared CM plus psychotherapy with each specific intervention. However, only the direct comparison between CM plus psychotherapy and psychotherapy was available, and the results suggested that CM plus psychotherapy revealed a statistically higher rate of alcohol abstinence (OR, 2.277 [95% CI, 1.271–4.081]) in treatment sessions (Table S5 in supporting information). In follow-up sessions, pharmacotherapy plus psychotherapy was directly compared with 2 other interventions: pharmacotherapy plus BI and pharmacotherapy. With direct comparison, we observed that pharmacotherapy plus psychotherapy showed significant differences in alcoholic abstinence rates compared to psychotherapy alone (OR, 1.410 [95% CI, 1.005–1.978]) but not pharmacotherapy plus BI (OR, 1.302 [95% CI, 0.830–2.041]) (Table S5 in supporting information).

In summary, based on all these results of pairwise comparisons, we demonstrated that application of alcoholic interventions could significantly enhance abstinence rates. In addition, in total, the abovementioned 2 superior interventions were verified to be better than other interventions. However, these direct pairwise comparisons did not cover all interventions; thus, the validation of the main results was not totally completed.

### 3.5 Publication bias and data consistency

We did not observe any evidence of publication bias in either treatment sessions or follow-up sessions according to funnel-plot asymmetry after quantitative calculations (Figure S5 in supporting information). Additionally, to test network coherence, the differences between the direct and indirect effects in the closed loops were estimated by a node-splitting model. This approach was used to access the network inconsistency and coherence. No significant difference was detected in either the treatment session or in the follow-up session after assessment (*P* > .05 for all) (Table S6 in the supporting information). Based on all these results, we may conclude that our results did not reveal obvious publication bias and exhibited good consistency.

### 3.6 Quality of evidence

There were 20 and 16 direct comparisons for treatment sessions and follow-up sessions, respectively. In addition, both of them had 36 possible comparisons in the network. On applying GRADE to findings from the network meta-analysis combining direct and indirect evidence, the quality of evidence for treatment sessions was 19/36 (53%), which was classified as high or moderate. In addition, for follow-up sessions, only 6 (17%) comparisons were rated as high or moderate (Table S7 in the supporting information).

## 4 Discussion

In the present study, direct and indirect evidence from 137 RCTs containing 27,282 participants with AUDs was analyzed to elucidate the association of various alcoholic interventions with abstinence rates in treatment sessions and follow-up sessions based on Bayesian network meta-analysis. We demonstrated that alcoholic interventions were effective in both treatment sessions and follow-up sessions. In treatment sessions, the application of CM plus psychotherapy was associated with the highest probability of achieving the highest abstinence rate with a good quality of evidence. On the other hand, pharmacotherapy plus psychotherapy was associated with the highest abstinence rate in follow-up sessions, yet the recommendation of evidence was limited. Additionally, the applications of CM plus psychotherapy and pharmacotherapy plus psychotherapy were proven to have higher abstinence rates than other interventions at the statistically significant level. For the 1st time, although we quantitatively analyzed the association of various alcoholic interventions with abstinence rates, the objective results still need to be further explained and discussed.

In treatment sessions, CM plus psychotherapy was verified to be the best intervention, associated with the highest alcohol abstinence rate, followed by CM. The CM is an intervention in which reinforcers, such as vouchers or prizes, are provided, typically multiple times per week, when individuals demonstrate substance abstinence.^[31]^ Petry et al 1st demonstrated that CM was associated with higher alcoholic abstinence rates,^[32]^ and this approach was demonstrated to be effective in the treatment of AUDs in the following years.^[33–34]^ The CM was developed based on psychological therapy,^[35]^ and some psychiatrists refer to CM as part of psychotherapy.^[36]^ However, performing CM is directly aiming at abstinence, which is triggered by goods or money. This approach does not need complicated systematic psychotherapy, and the participants do not even need to meet the conductors,^[37]^ who may not even need to be professional psychologists either. We deemed CM an independent approach, which was the only “yes or no” alcoholic treatment procedure directly focusing on abstinence, and CM plus psychotherapy was proven to be associated with the highest probability of achieving abstinence. Here, speaking of psychotherapy, which focuses on motivational and behavioral switches, the results of the direct meta-analysis showed that psychotherapy alone did not confer any benefit to alcoholic abstinence compared to controls (Table S4 in the supporting information). Meanwhile, previous RCTs have illustrated that psychotherapy may ameliorate alcoholism regarding some subjective parametric data but exhibited no significant benefit in achieving or maintaining higher abstinence rates.^[38–41]^ In addition, evidence-based medicine also proved that there was no substantial enhancement of abstinence using psychotherapy.^[42–43]^ Thereby, we may conclude that CM was the key factor in achieving or maintaining the highest abstinence rates in treatment sessions.

On the other hand, for the outcomes of follow-up sessions, we determined that pharmacotherapy plus psychotherapy seemed to have the best chance of achieving the highest abstinence rate. Pharmacotherapy, which aims to lessen the physiological dependence for AUDs, was approved by the FDA and had been demonstrated to bring significant benefits to reduce alcoholism to maintain abstinence and prevent relapse.^[13–14,44–47]^ Additionally, the direct meta-analysis in the present study concluded that pharmacotherapy was the only intervention that may increase the abstinence rate in follow-up sessions (Table S4 in the supporting information). Although pharmacotherapy plus psychotherapy possessed the best rank based on network comparison, direct meta-analysis exhibited that pharmacotherapy plus psychotherapy revealed no significant difference compared to pharmacotherapy plus BI (Table S5 in the supporting information). As mentioned above, psychotherapy conferred no benefit to abstinence, and a large sample RCT reported by Anton et al^[48]^ also concluded that pharmacotherapy may show similar efficacy on abstinence with or without psychotherapy. Moreover, BI was confirmed to validate the achievement of higher abstinence in treatment sessions (Table S4 in the supporting information),^[49–51]^ yet it was also determined to have no efficacy on post-treatment session.^[52–53]^ Thus, pharmacotherapy plus psychotherapy revealed similar benefits compared with pharmacotherapy plus BI. Taken together, these findings indicate that we regard pharmacotherapy as the crucial and maybe the only factor of achieving the highest alcoholic abstinence rates in follow-up sessions.

In summary, by network quantitative analysis, we concluded that CM plus psychotherapy possessed the best rank of achieving the highest abstinence rate in treatment sessions, and pharmacotherapy plus psychotherapy possessed the best rank in follow-up sessions. Despite the fact that psychotherapy was involved in the best rank in both sessions, we confirmed that it may not have relative efficacy. Meanwhile, it should be noticed that pharmacotherapy was the only intervention that was statistically confirmed as effective in both sessions (Table S4 and Table S5 in the supporting information). The CM revealed great efficacy in treatment sessions, but we do not have enough direct statistical evidence to estimate CM in follow-up sessions, although CM-related interventions were similar to pharmacotherapy-related sessions in maintaining the highest abstinence rates in follow-up sessions (Fig. 4). More importantly, based on our evidence, we may discover that economics seemed to be the strongest motivational stimulus for changing drinking behavior, and simultaneously, pharmacotherapy was an effective adjuvant physiological therapy for its long-term effectiveness. Therefore, we may raise the following questions: would the combination of CM and pharmacotherapy be associated with the highest abstinence rates in future alcoholic treatment? Should pharmacotherapy be viewed as basic treatment? We expect more RCTs to examine these questions in the future.

After analyzing and explaining our findings, we admit some limitations in the current meta-analysis. First, since the funnel-plot and node-splitting model did not detect obvious publication bias or any data inconsistency, most of the included trials were conducted in developed countries with population diversity; thus, some conceptual heterogeneity may exist and contribute to our results. Second, despite the fact that 35 trials with 6329 participants were included, the quality of evidence of follow-up sessions was low. This issue may introduce confounding factors into our analysis. Moreover, the raw data for direct meta-analysis was insufficient to cover all included interventions, and a paucity of some pairwise comparisons existed (e.g., CM plus psychotherapy versus CM alone), so some conclusions could not be further confirmed, and the validation of direct comparisons could not be fully completed. Finally, ranking probabilities may be influenced by unequal numbers of trials per comparison and network configuration. For these reasons, these results require further statistical validation and should be interpreted with caution.

In summary, the application of alcoholic interventions was effective to achieve and maintain abstinence rates. In addition, among these interventions, contingency management plus psychotherapy and pharmacotherapy plus psychotherapy were demonstrated to be associated with highest abstinence rates in treatment sessions and follow-up sessions, respectively. In addition, contingency management and pharmacotherapy seemed to be the key factors affecting alcoholic abstinence in treatment sessions and follow-up sessions, respectively. However, this conclusion still requires more evidence for further validation.

## Acknowledgment

The authors report no financial relationships with commercial interests.

## Author contributions

**Conceptualization:** Jiamin Gao.

**Data curation:** Jiamin Gao.

**Formal analysis:** Jiamin Gao.

**Funding acquisition:** Jiamin Gao.

**Investigation:** Jiamin Gao, Jun Cao.

**Methodology:** Jiamin Gao.

**Project administration:** Jiamin Gao, Yunyue Xiao.

**Resources:** Jiamin Gao, Jun Cao.

**Software:** Jiamin Gao, Jun Cao.

**Supervision:** Jun Cao, Tao Guo, Yunyue Xiao.

**Validation:** Jun Cao, Tao Guo.

**Visualization:** Jun Cao, Tao Guo.

**Writing – original draft:** Jun Cao, Tao Guo.

**Writing – review & editing:** Tao Guo, Yunyue Xiao.

## Supplementary Material

Supplemental Digital Content
