# Supplementary material for: Association between alcoholic interventions and abstinence rates for alcohol use disorders: A meta-analysis
Source: Medicine (Baltimore). 2018 Dec 14;97(50):e13566. doi: 10.1097/MD.0000000000013566 (PMC6320082; doi:10.1097/MD.0000000000013566)
Supplement: Supplemental Digital Content [file medi-97-e13566-s001.pdf]

Figure S1. Quality assessment of (A) overall and (B) study-level risk of bias, using Cochrane's risk of bias assessment tool, indicating quality with high ratings, unclear or low risk of bias, according to 6 evaluation criteria for each study, as presented in detail.

Figure S1A.

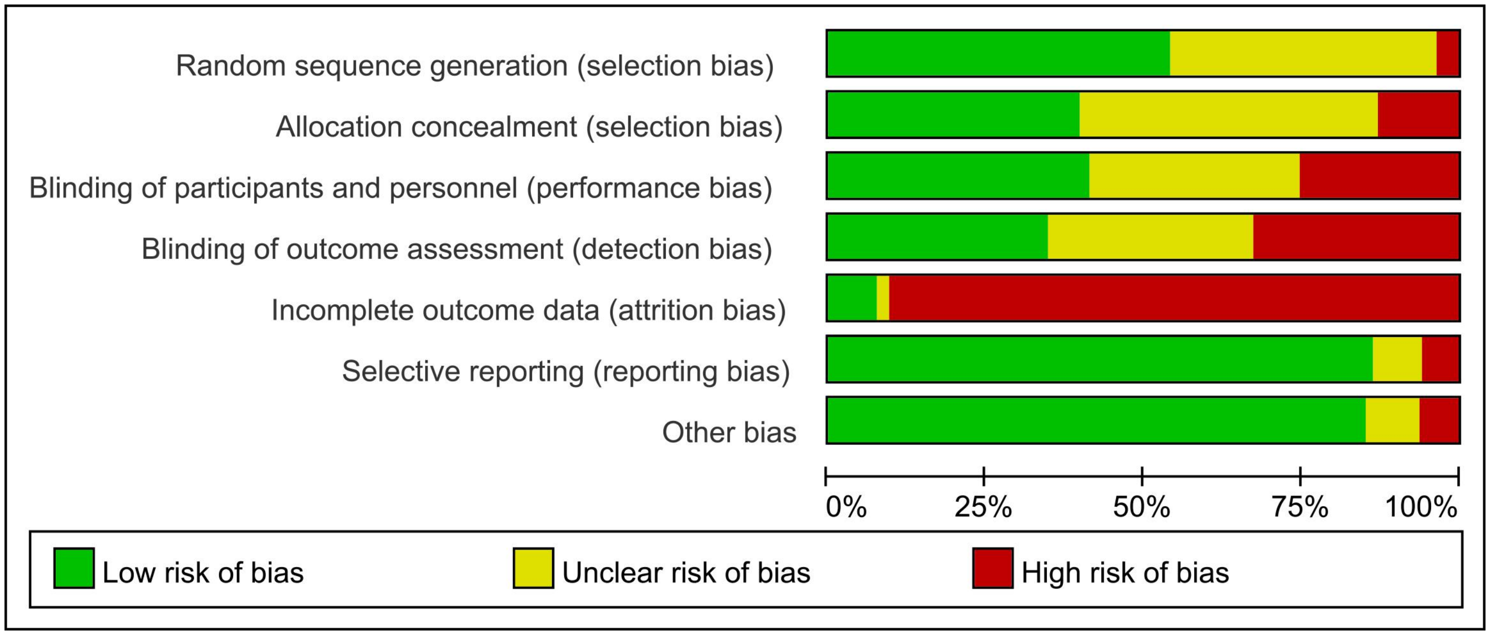

Figure S1B.

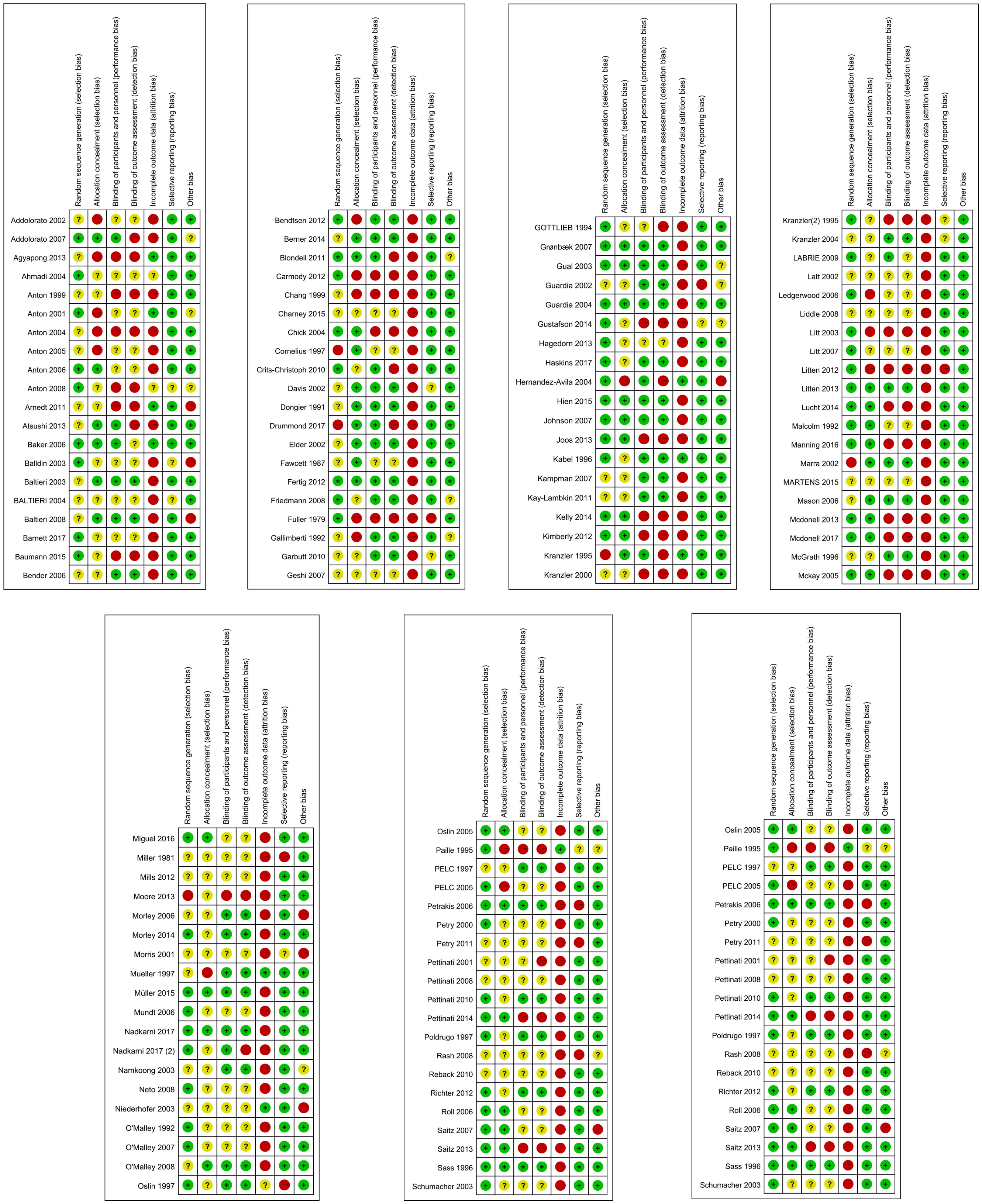
Random sequence generation (selection bias)
Allocation concealment (selection bias)
Blinding of participants and personnel (performance bias)
Blinding of outcome assessment (detection bias)
Incomplete outcome data (attrition bias)
Selective reporting (reporting bias)
Other bias
Addolorato 2002
Addolorato 2007
Agyapong 2013
Ahmadi 2004
Anton 1999
Anton 2001
Anton 2004
Anton 2005
Anton 2006
Anton 2008
Arnedt 2011
Atsushi 2013
Baker 2006
Balldin 2003
Baltieri 2003
BALTIERI 2004
Baltieri 2008
Barnett 2017
Baumann 2015
Bender 2006
Bendtsen 2012
Berner 2014
Blondell 2011
Carmody 2012
Chang 1999
Charney 2015
Chick 2004
Cornelius 1997
Crits-Christoph 2010
Davis 2002
Dongier 1991
Drummond 2017
Elder 2002
Fawcett 1987
Fertig 2012
Friedmann 2008
Fuller 1979
Gallimberti 1992
Garbutt 2010
Geshi 2007
GOTTLIEB 1994
Grønbæk 2007
Gual 2003
Guardia 2002
Guardia 2004
Gustafson 2014
Hagedorn 2013
Haskins 2017
Hernandez-Avila 2004
Hien 2015
Johnson 2007
Joos 2013
Kabel 1996
Kampman 2007
Kay-Lambkin 2011
Kelly 2014
Kimberly 2012
Kranzler 1995
Kranzler 2000
Kranzler(2) 1995
Kranzler 2004
LABRIE 2009
Latt 2002
Ledgerwood 2006
Liddle 2008
Litt 2003
Litt 2007
Litten 2012
Litten 2013
Lucht 2014
Malcolm 1992
Manning 2016
Marra 2002
MARTENS 2015
Mason 2006
Mcdonell 2013
Mcdonell 2017
McGrath 1996
Mckay 2005
Miguel 2016
Miller 1981
Mills 2012
Moore 2013
Morley 2006
Morley 2014
Morris 2001
Mueller 1997
Müller 2015
Mundt 2006
Nadkarni 2017
Nadkarni 2017 (2)
Namkoong 2003
Neto 2008
Niederhofer 2003
O'Malley 1992
O'Malley 2007
O'Malley 2008
Oslin 1997
Oslin 2005
Paille 1995
PELC 1997
PELC 2005
Petrakis 2006
Petry 2000
Petry 2011
Pettinati 2001
Pettinati 2008
Pettinati 2010
Pettinati 2014
Poldrugo 1997
Rash 2008
Reback 2010
Richter 2012
Roll 2006
Saitz 2007
Saitz 2013
Sass 1996
Schumacher 2003

Figure S2. Network connections of included studies with the available direct comparisons for abstinence rates regarding (A) treatment sessions and (B) follow-up sessions. The details of sample size and trial numbers were presented.

Figure S2A.

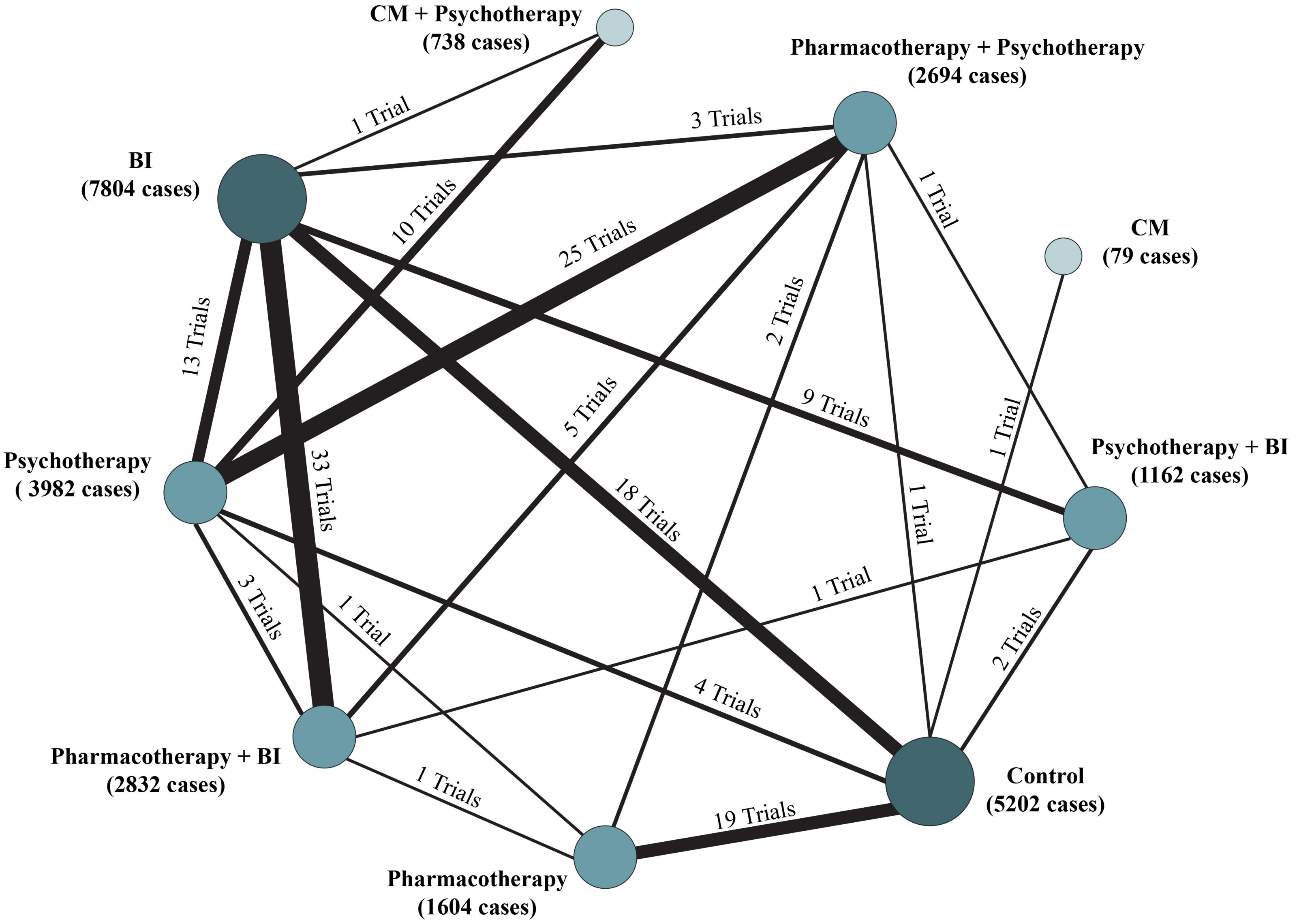

The size of circles and width of lines respectively represent the number of included sample sizes and trails. CM, contingency management; BI, Brief Intervention.

- **≥ 5000 cases**
- **≥ 1000 and < 5000 cases**
- **< 1000 cases**

Figure S2B.

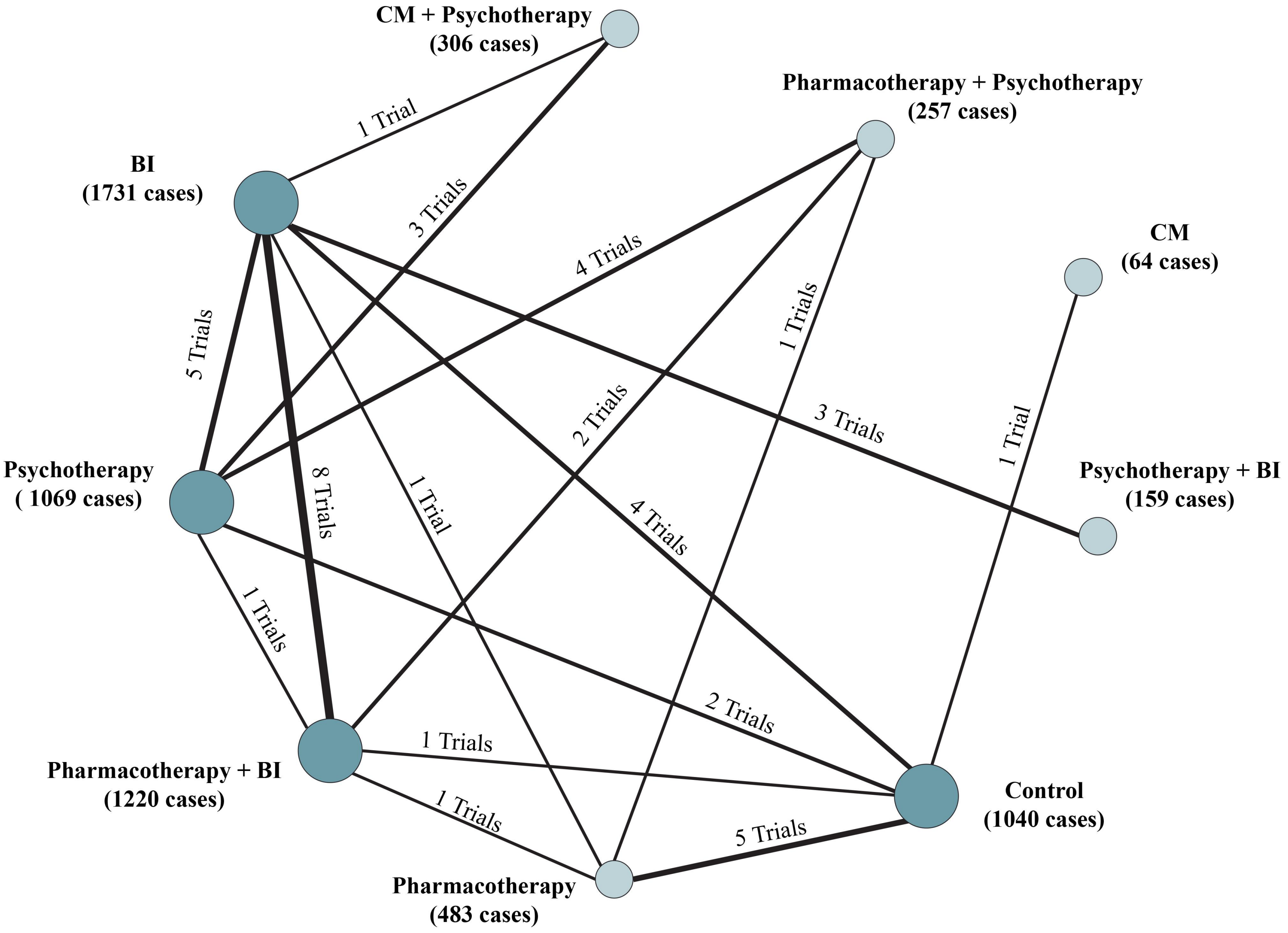

The size of circles and width of lines respectively represent the number of included sample sizes and trails. CM, contingency management; BI, Brief Intervention.

≥ 1000 and < 5000 cases

< 1000 cases

Figure S3. Direct meta-analysis of interventions versus control regarding the abstinence rates of (A) treatment sessions and (B) follow-up sessions. The details were consistently exhibited in Table S6.

Figure S3A.

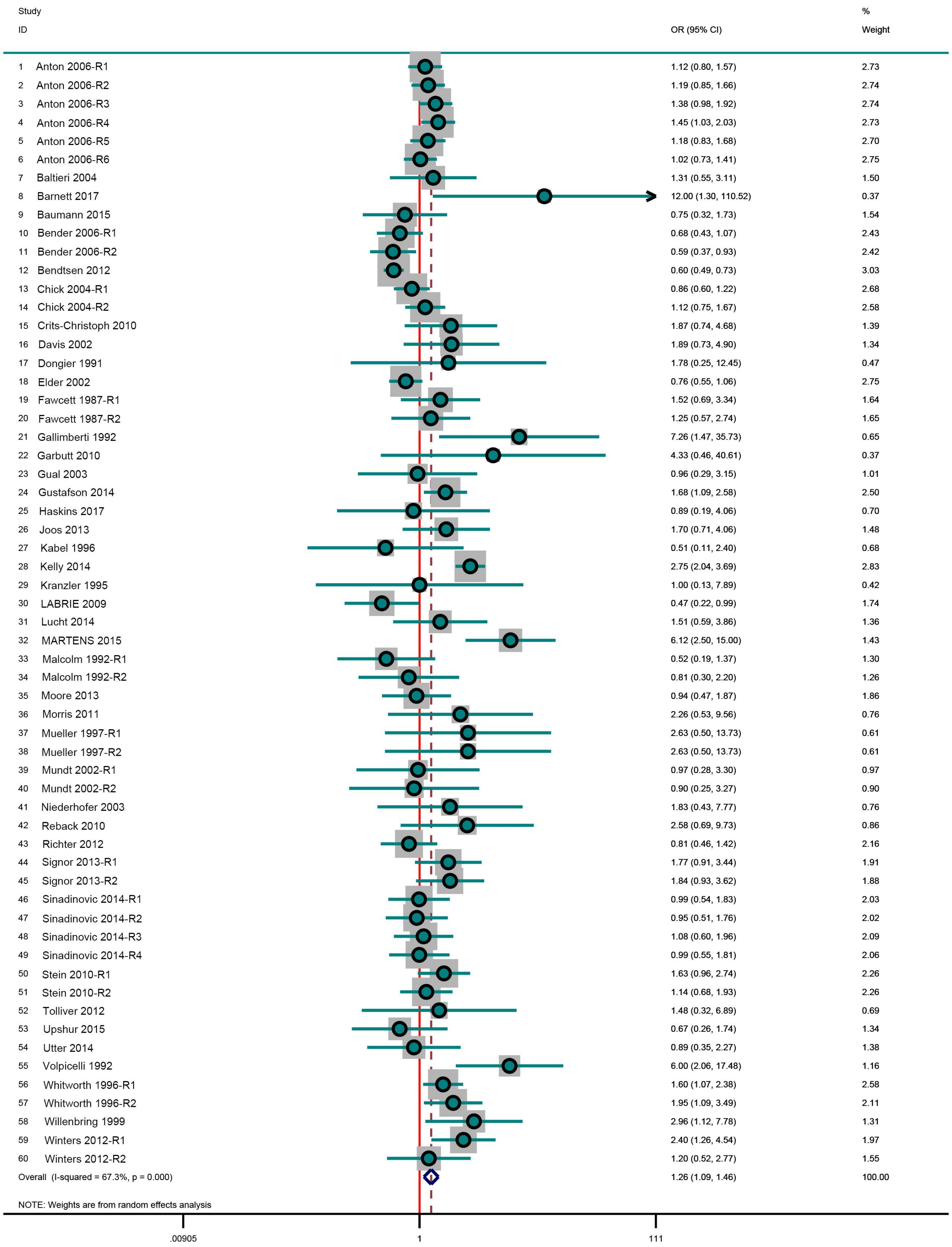

Figure S3B.

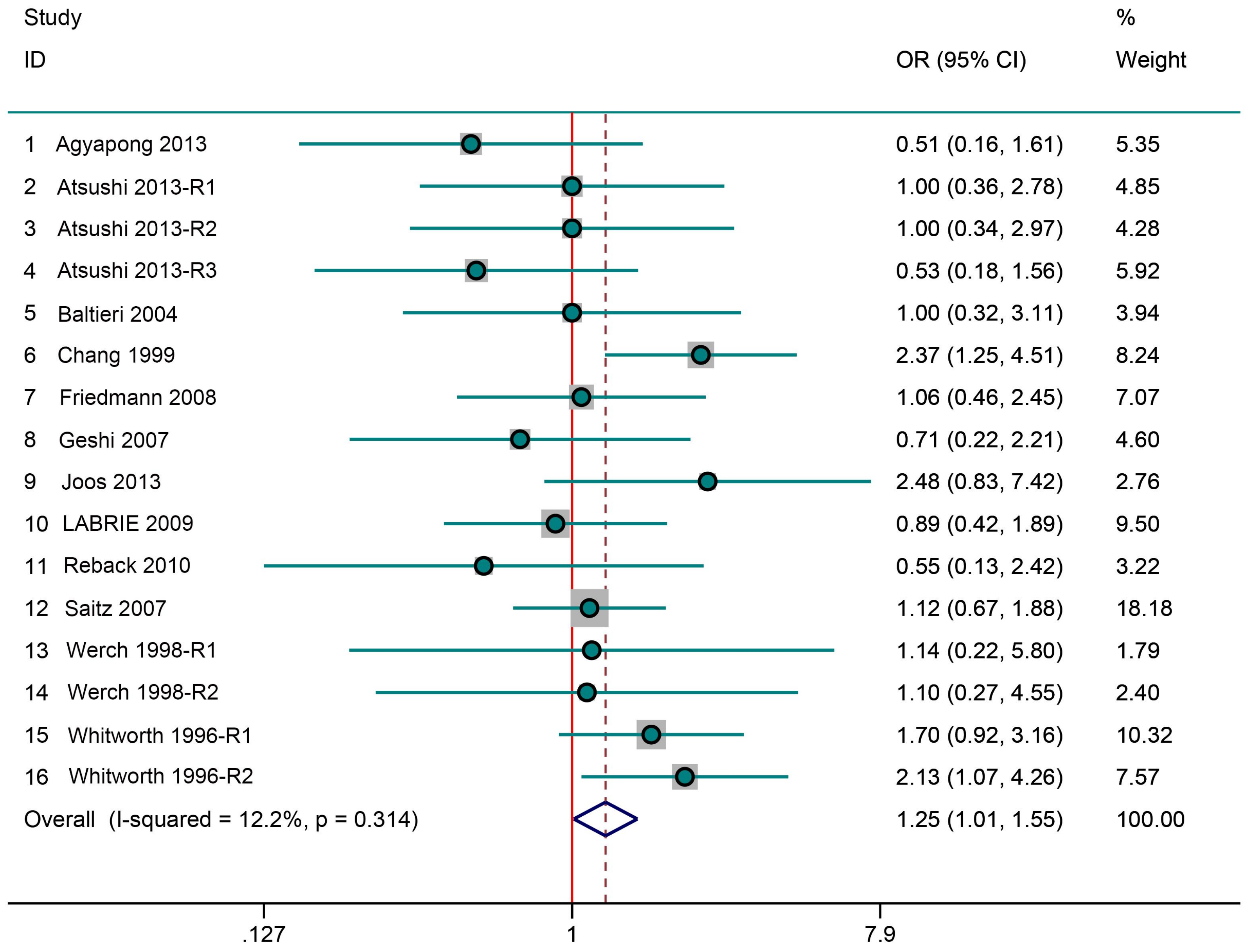
Study
ID
OR (95% CI)
%
Weight
1 Agyapong 2013 0.51 (0.16, 1.61) 5.35
2 Atsushi 2013-R1 1.00 (0.36, 2.78) 4.85
3 Atsushi 2013-R2 1.00 (0.34, 2.97) 4.28
4 Atsushi 2013-R3 0.53 (0.18, 1.56) 5.92
5 Baltieri 2004 1.00 (0.32, 3.11) 3.94
6 Chang 1999 2.37 (1.25, 4.51) 8.24
7 Friedmann 2008 1.06 (0.46, 2.45) 7.07
8 Geshi 2007 0.71 (0.22, 2.21) 4.60
9 Joos 2013 2.48 (0.83, 7.42) 2.76
10 LABRIE 2009 0.89 (0.42, 1.89) 9.50
11 Reback 2010 0.55 (0.13, 2.42) 3.22
12 Saitz 2007 1.12 (0.67, 1.88) 18.18
13 Werch 1998-R1 1.14 (0.22, 5.80) 1.79
14 Werch 1998-R2 1.10 (0.27, 4.55) 2.40
15 Whitworth 1996-R1 1.70 (0.92, 3.16) 10.32
16 Whitworth 1996-R2 2.13 (1.07, 4.26) 7.57
Overall (I-squared = 12.2%, p = 0.314) 1.25 (1.01, 1.55) 100.00
.127
1
7.9

Figure S4. Direct meta-analysis of best rank intervention versus other interventions regarding the abstinence rates of (A) treatment sessions and (B) follow-up sessions. The detailed overall and subgroup effects were also exhibited in Table S7.

Figure S4A.

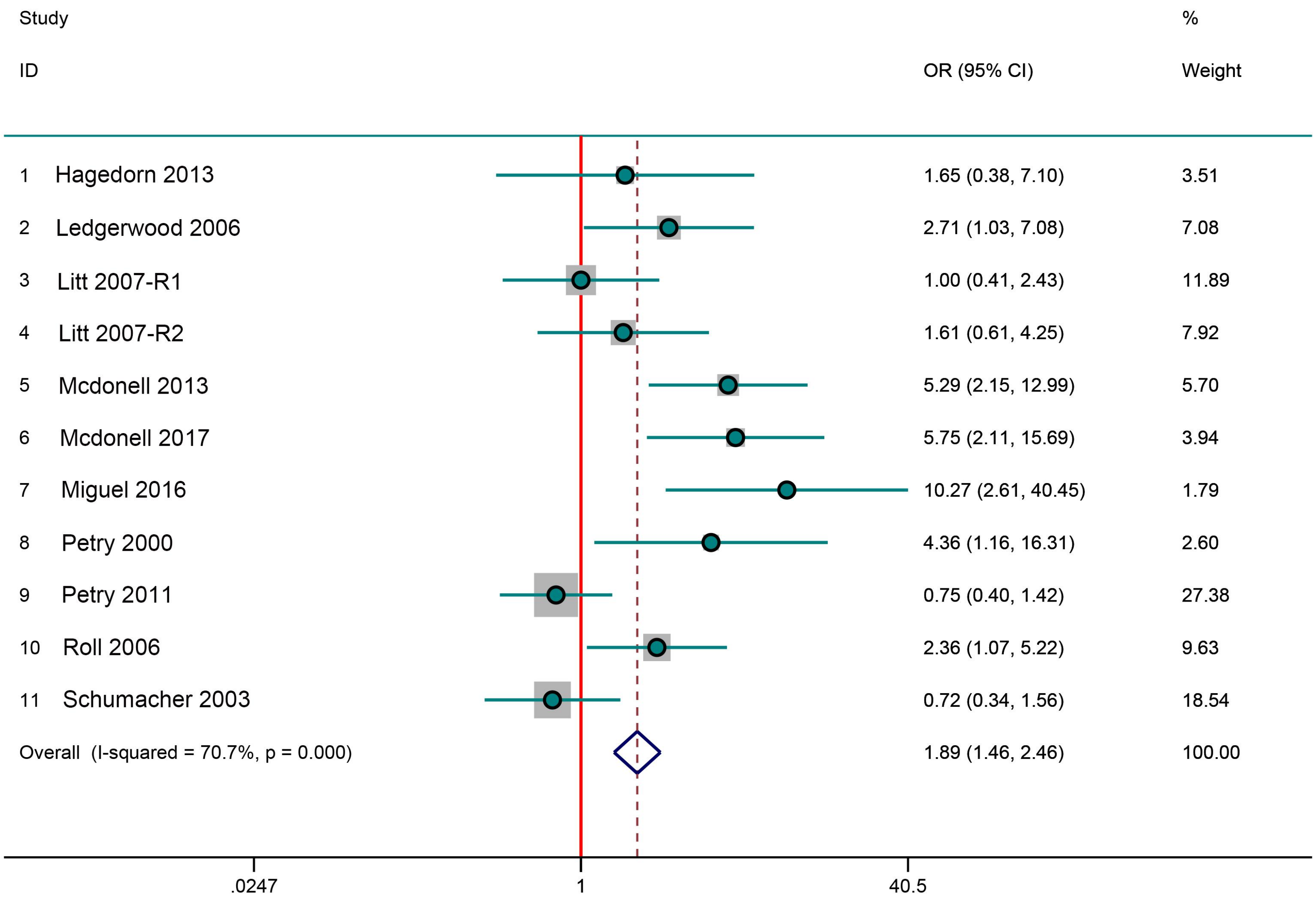

Figure S4B.

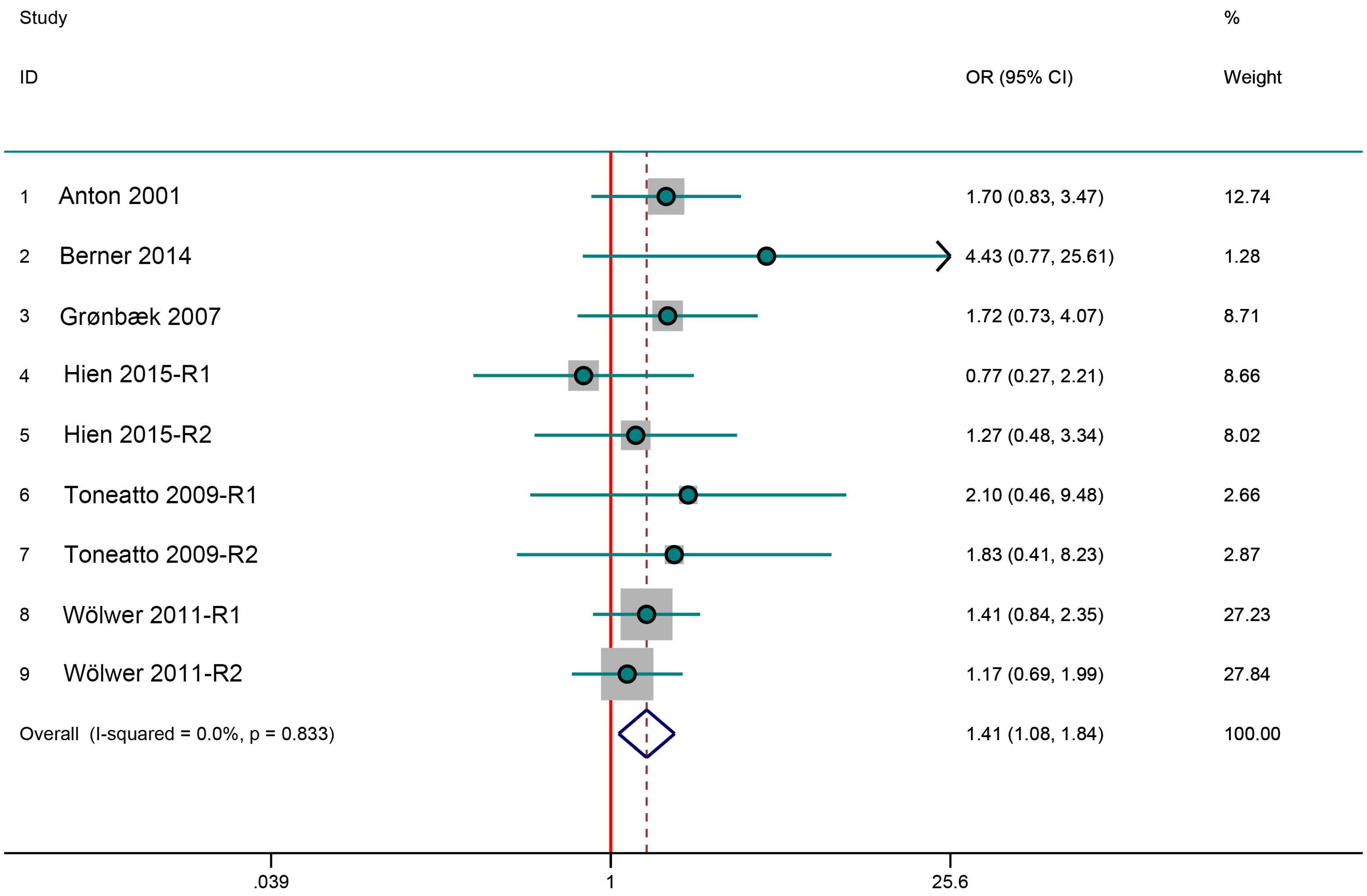
Study
ID
OR (95% CI)
%
Weight
1 Anton 2001
1.70 (0.83, 3.47)
12.74
2 Berner 2014
4.43 (0.77, 25.61)
1.28
3 Grønbæk 2007
1.72 (0.73, 4.07)
8.71
4 Hien 2015-R1
0.77 (0.27, 2.21)
8.66
5 Hien 2015-R2
1.27 (0.48, 3.34)
8.02
6 Toneatto 2009-R1
2.10 (0.46, 9.48)
2.66
7 Toneatto 2009-R2
1.83 (0.41, 8.23)
2.87
8 Wölwer 2011-R1
1.41 (0.84, 2.35)
27.23
9 Wölwer 2011-R2
1.17 (0.69, 1.99)
27.84
Overall (I-squared = 0.0%, p = 0.833)
1.41 (1.08, 1.84)
100.00
.039
1
25.6

Figure S5. Publication bias of studies on abstinence rates regarding **(A)** treatment sessions and **(B)** follow-up sessions was assessed by funnel plots. All studies are centered on the summary effect estimate of their respective comparisons [μXY (logOR for the present study)], which is represented by the vertical red line.

Figure S5A.

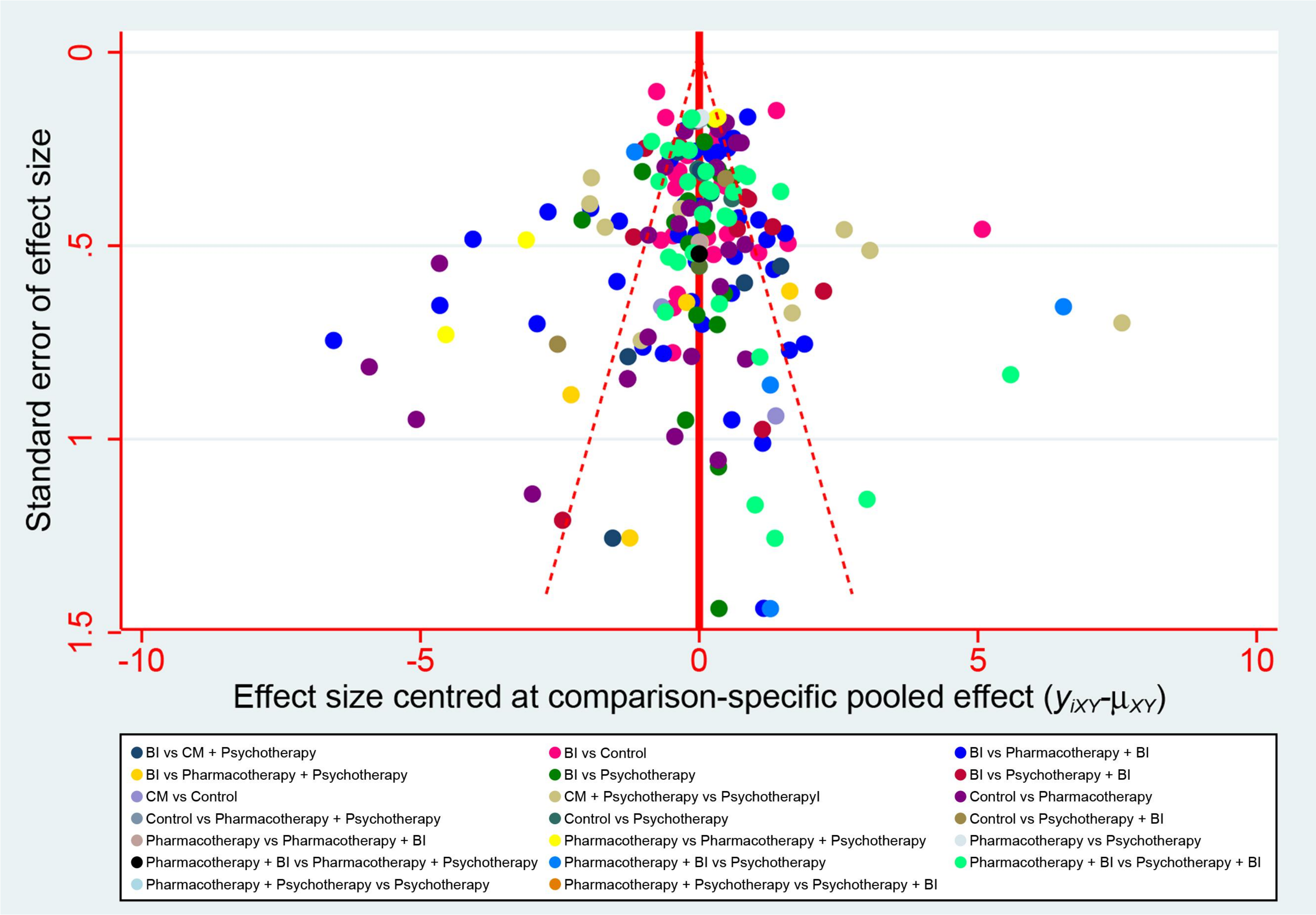

Figure S5B.

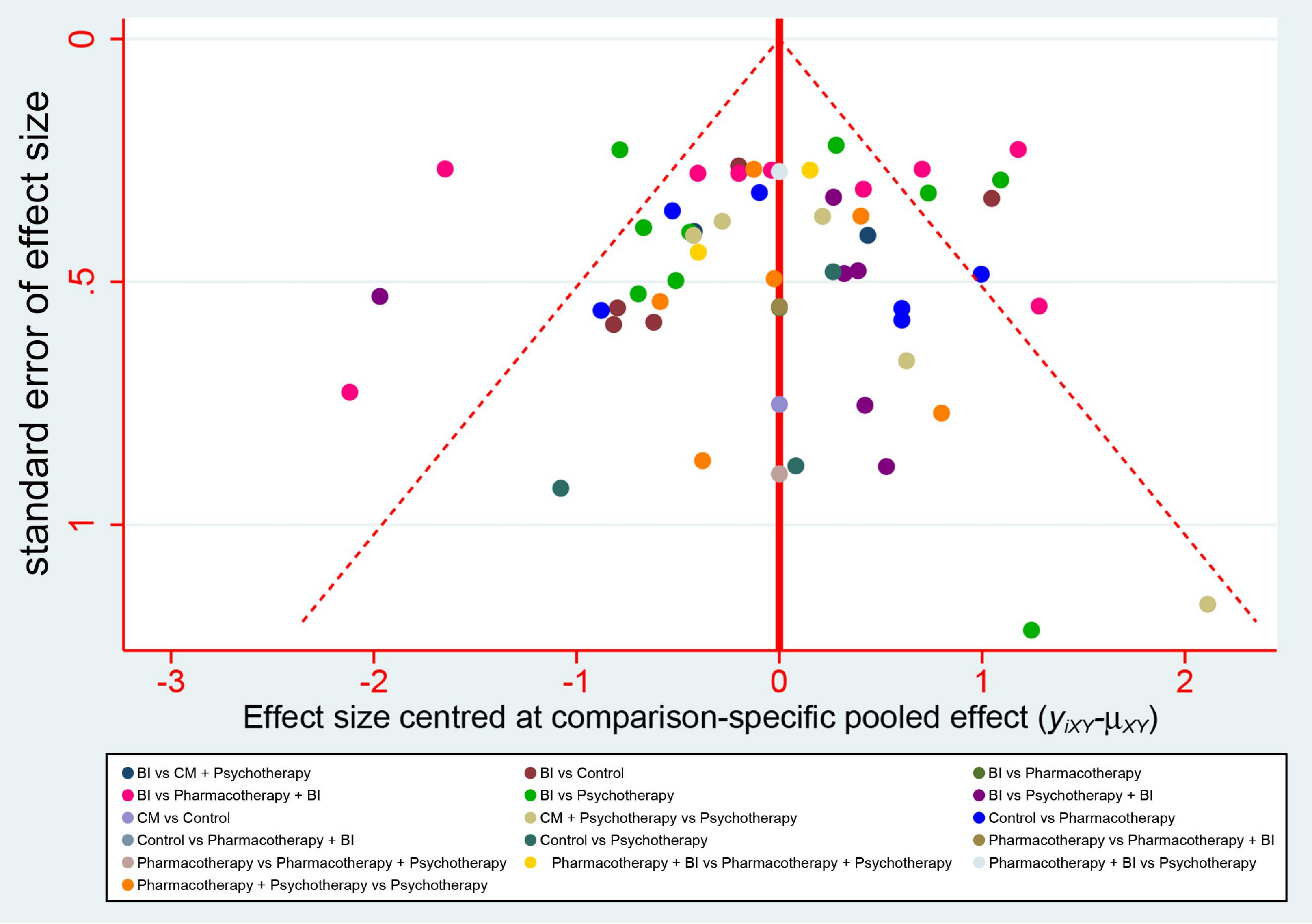

0
.5
1
standard error of effect size
-3
-2
-1
0
1
2
Effect size centred at comparison-specific pooled effect (y_{iXY}-μ_{XY})
BI vs CM + Psychotherapy
BI vs Control
BI vs Pharmacotherapy
BI vs Pharmacotherapy + BI
BI vs Psychotherapy
BI vs Psychotherapy + BI
CM vs Control
CM + Psychotherapy vs Psychotherapy
Control vs Pharmacotherapy
Control vs Pharmacotherapy + BI
Control vs Psychotherapy
Pharmacotherapy vs Pharmacotherapy + BI
Pharmacotherapy vs Pharmacotherapy + Psychotherapy
Pharmacotherapy + BI vs Pharmacotherapy + Psychotherapy
Pharmacotherapy + BI vs Psychotherapy
Pharmacotherapy + Psychotherapy vs Psychotherapy

Table S1. Databases: MEDLINE. Citations were taken from 1964 to present (No publication status restriction).

# No.	Search	Results
1	exp (alcohol) AND ((consumption) OR abuse) OR dependence) OR disorder)/	4767213
2	exp binge drinking/	5080
3	exp excessive drinking/	3996
4	1 OR 2 OR 3	4796352
5	(brief or brief intervention or BI). ti,ab.	1011824
6	5 AND 4	170724
7	(mental intervention or psychological intervention or psychological support). ti,ab.	4170079
8	(motivational interviewing or MI or motivational enhancement therapy or MET). ti,ab.	1565463
9	(cognitive behavioral therapy or CBT or coping skills intervention). ti,ab.	117864
10	(TSF or 12-Step Facilitation). ti,ab.	1286
11	(therapeutic community or TC or family therapy). ti,ab.	6956
12	(vocational rehabilitation or VR). ti,ab.	2355
13	7 OR 8 OR 9 OR 10 OR 11 OR 12	2025513
14	13 AND 4	578426
15	(pharmacotherapy or pharmacological intervention or medical intervention). ti,ab.	4684189
16	((5-HT (serotonin) partial agonists OR anticonvulsants OR antipsychotics OR benzodiazepines OR beta-blockers OR monoamine oxidase inhibitors (MAOIs) OR noradrenaline re-uptake inhibitors (NARIs) OR noradrenergic and specific serotonergic antidepressants (NaSSAs) OR reversible inhibitors of monoamine oxidase A (RIMAs) OR serotonin antagonist and re-uptake inhibitors (SARIs) OR serotonin and noradrenaline re-uptake inhibitors (SNRIs) OR selective serotonin re-uptake inhibitors (SSRIs) OR tricyclic antidepressants (TCAs))). ti,ab.	11533
17	15 OR 16	4684442

18	17 AND 4	1298644
19	(acupuncture or moxibustion). ti,ab.	28011
20	19 AND 4	13938
21	(contingency management or CM or behavioral economic supplement). ti,ab.	350499
22	21 AND 4	71333
23	(random or prospective). ti,ab.	1134069
24	(randomized controlled trial). ti,ab.	589286
25	23 OR 24	1775771
26	6 OR 14 OR 18 OR 20 OR 22	7256376
27	25 AND 26	3265426
28	human.sh	17613440
29	animal.sh.	6343795
30	29 NOT 28	5214790
31	27 NOT 30	42369

Table S2. General information about the included RCTs. [BI: Brief Intervention; CM: Contingency Management; √: Marked as Available Data]

Author	Time of Pub.	Country	Study Arms	Interventional Application					Sample Size	Abstinent Rate	
				BI	CM	Pharmacotherapy	Psychotherapy	Control		Treatment Session	Follow-up Session
Addolorato	2002	Italy	2	√		√			39	√	
Addolorato	2007	Italy	2	√		√			84	√	
Agyapong	2013	Ireland	2	√				√	48	√	√
Ahmadi	2004	Iran	2	√		√			116	√	
Anton	1999	USA	2			√	√		131	√	
Anton	2001	USA	2			√	√		131	√	√
Anton	2004	USA	2			√	√		270	√	
Anton	2005	USA	4			√	√		160	√	
Anton	2006	USA	4			√	√	√	1226	√	
Anton	2008	USA	2			√	√		288	√	
Arnedt	2011	USA	2	√			√		17	√	
Atsushi	2014	Japan	4	√		√		√	109		√
Baker	2006	Australia	2	√			√		54	√	√
Balldin	2003	Sweden	4	√		√	√		118	√	
Baltieri	2003	Brazil	2	√		√			75	√	√
Baltieri	2004	Brazil	2			√		√	75	√	√
Baltieri	2008	Brazil	2	√		√			155	√	
Barnett	2017	USA	2		√			√	29	√	
Baumann	2015	Germany	2	√				√	629	√	
Bender	2006	Germany	2			√		√	299	√	
Bendtsen	2012	Sweden	2	√				√	2336	√	
Berner	2014	Germany	2			√	√		96	√	√
Blondell	2011	USA	2				√	√	138	√	
Carmody	2012	USA	2				√	√	162	√	√
Chang	1999	USA	2	√				√	250		√
Charney	2015	Canada	2			√	√		265	√	
Chick	2004	UK	2			√		√	492	√	
Cornelius	1997	USA	2	√		√			51	√	
Crits-Christoph	2010	USA	2	√				√	118	√	
Davis	2002	USA	2	√				√	84	√	
Dongier	1991	Canada	2			√		√	23	√	
Drummond	2017	UK	2	√			√		94	√	
Elder	2002	USA	2	√				√	630	√	
Fawcett	1987	USA	2			√		√	104	√	
Fertig	2012	USA	2	√		√			130	√	
Friedmann	2008	USA	2			√		√	173		√
Fuller	1979	USA	2	√		√			128	√	
Gallimberti	1992	Italy	2			√		√	71	√	
Garbutt	2010	USA	2			√		√	80	√	

Geshi	2007	Japan	2	√				√	71		√
GOTTLIEB	1994	USA	2	√		√			100	√	
Grønbæk	2007	Denmark	2	√		√	√		87		√
Gual	2003	Spain	2			√		√	83	√	
Guardia	2002	Spain	2	√		√			202	√	
Guardia	2004	Spain	2			√	√		60	√	
Gustafson	2014	USA	2	√				√	349	√	
Hagedorn	2013	USA	2		√		√		191	√	√
Haskins	2017	USA	2	√				√	212	√	
Hernandez-Avila	2004	USA	2	√		√			41	√	
Hien	2015	USA	2			√	√		69	√	√
Johnson	2007	USA	2			√	√		364	√	
Joos	2013	Belgium	2			√		√	83	√	√
Kabel	1996	USA	2			√		√	28	√	
Kampman	2007	USA	2			√	√		61	√	
Kay-Lambkin	2011	Australia	2	√			√		274	√	
Kelly	2014	Kenya	2	√				√	752	√	
Kimberly	2012	USA	2	√			√		117	√	
Kranzler	1995	USA	2			√		√	40	√	
Kranzler (2)	1995	USA	2			√	√		94	√	
Kranzler	2000	USA	2			√	√		183	√	
Kranzler	2004	USA	2			√	√		315	√	
LABRIE	2009	USA	2				√	√	285	√	√
Latt	2002	Australia	2	√		√			107	√	
Ledgerwood	2006	USA	2		√		√		142	√	
Liddle	2008	USA	2	√			√		224	√	√
Litt	2003	USA	2	√			√		128	√	√
Litt	2007	USA	3	√	√		√		195	√	√
Litten	2012	USA	2	√		√			215	√	
Litten	2013	USA	2	√		√			197	√	
Lucht	2014	Germany	2	√				√	80	√	
Malcolm	1992	USA	2			√		√	67	√	
Manning	2016	Australia	2	√			√		83	√	
Marra	2002	France	2	√		√			31	√	
MARTENS	2015	USA	2	√				√	325	√	
Mason	2006	USA	2	√		√			592	√	
Mcdonell	2013	USA	2		√		√		176	√	
Mcdonell	2017	USA	2		√		√		79	√	
McGrath	1996	USA	2	√		√			56	√	
Mckay	2005	USA	2	√			√		359	√	√
Miguel	2016	Brazil	2		√		√		65	√	
Miller	1981	USA	2	√			√		31	√	√

Mills	2012	Australia	2	√			√		103	√	√
Moore	2013	UK	2	√				√	554	√	
Morley	2006	Australia	2	√		√			94	√	
Morley	2014	Australia	2			√	√		42	√	
Morris	2001	Australia	2			√		√	71	√	
Mueller	1997	USA	2			√		√	27	√	
Müller	2015	Germany	2	√		√			56	√	√
Mundt	2006	USA	3	√				√	60	√	
Nadkarni	2017	India	2	√			√		336	√	
Nadkarni (2)	2017	India	2	√			√		316	√	
Namkoong	2003	South Korea	2			√	√		142	√	
Neto	2008	Portugal	2	√			√		209	√	
Niederhofer	2003	Austria	2			√		√	26	√	
O’Malley	1992	USA	3	√		√	√		104	√	
O’Malley	2007	Ireland	2			√	√		93	√	
O’Malley	2008	USA	2	√		√			101	√	
Oslin	1997	USA	2	√		√			44	√	
Oslin	2005	USA	2	√		√			74	√	
Paille	1995	France	2	√		√			438	√	√
PELC	1997	Belgium	2	√		√			188	√	
PELC	2005	France	2	√		√			100	√	
Petrakis	2006	USA	2			√	√		254	√	
Petry	2000	USA	2		√		√		42	√	
Petry	2011	USA	2		√		√		244	√	
Pettinati	2001	USA	2	√		√			100	√	
Pettinati	2008	USA	2			√	√		208	√	
Pettinati	2010	USA	2			√	√		170	√	
Pettinati	2014	USA	2			√	√		80	√	
Poldrugo	1997	Italy	2	√		√			246	√	√
Rash	2008	USA	2		√		√		208		√
Reback	2010	USA	2		√			√	131	√	√
Richter	2012	Germany	2			√		√	201	√	
Roll	2006	USA	2		√		√		113	√	
Saitz	2007	USA	2	√				√	287		√
Saitz	2013	USA	2	√			√		532	√	
Sass	1996	Germany	2	√		√			272	√	√
Schumacher	2003	UK	2		√		√		127	√	
Signor	2013	Brazil	2	√				√	147	√	
Sinadinovic	2014	Sweden	3	√			√	√	633	√	
Stein	2010	USA	2	√				√	245	√	
Stephanie	2003	USA	4	√		√	√		113	√	
Tempesta	2000	Italy	2	√		√			329	√	√

Tolliver	2012	USA	2			√		√	33	√	
Toneatto	2009	Canada	2			√	√		52	√	√
Upshur	2015	USA	2	√				√	76	√	
Utter	2014	USA	2	√				√	181	√	
Volpicelli	1992	Canada	2			√		√	70	√	
Volpicelli	1997	Canada	2	√		√			97	√	
Werch	1998	USA	2				√	√	211	√	√
Whitworth	1996	Austria	2			√		√	448	√	√
Wiesbeck	2001	Germany	2	√		√			281	√	√
Wildt	2002	Netherland	2	√		√	√		241	√	
Willenbring	1999	USA	2	√				√	75	√	
Winters	2012	USA	2	√			√	√	315	√	
Winters	2014	USA	2	√			√	√	280	√	
Wölwer	2011	Germany	3	√		√	√		371	√	√

References

1. Addolorato G, Caputo F, Capristo E, et al. Baclofen efficacy in reducing alcohol craving and intake: a preliminary double-blind randomized controlled study. Alcohol Alcohol. 2002;37(5):504-8.
2. Addolorato G, Leggio L, Ferrulli A, et al. Effectiveness and safety of baclofen for maintenance of alcohol abstinence in alcohol-dependent patients with liver cirrhosis: randomised, double-blind controlled study. Lancet. 2007;370(9603):1915-22.
3. Agyapong VI, McLoughlin DM, Farren CK. Six-months outcomes of a randomised trial of supportive text messaging for depression and comorbid alcohol use disorder. J Affect Disord. 2013;151(1):100-4.
4. Ahmadi J, Babaeebeigi M, Maany I, et al. Naltrexone for alcohol-dependent patients. Ir J Med Sci. 2004;173(1):34-7.
5. Anton RF, Moak DH, Waid LR, et al. Naltrexone and cognitive behavioral therapy for the treatment of outpatient alcoholics: results of a placebo-controlled trial. Am J Psychiatry. 1999;156(11):1758-64.
6. Anton RF, Moak DH, Latham PK, et al. Posttreatment results of combining naltrexone with cognitive-behavior therapy for the treatment of alcoholism. J Clin Psychopharmacol. 2001;21(1):72-7.
7. Anton RF, Pettinati H, Zweben A, et al. A multi-site dose ranging study of nalmefene in the treatment of alcohol dependence. J Clin Psychopharmacol. 2004;24(4):421-8.
8. Anton RF, Moak DH, Latham P, et al. Naltrexone combined with either cognitive behavioral or motivational enhancement therapy for alcohol dependence. J Clin Psychopharmacol. 2005;25(4):349-57.
9. Anton RF, O'Malley SS, Ciraulo DA, et al. Combined pharmacotherapies and behavioral interventions for alcohol dependence: the COMBINE study: a randomized controlled trial. JAMA. 2006;295(17):2003-17.
10. Anton RF, Kranzler H, Breder C, et al. A randomized, multicenter, double-blind, placebo-controlled study of the efficacy and safety of aripiprazole for the treatment of alcohol dependence. J Clin Psychopharmacol. 2008;28(1):5-12.
11. Arnedt JT, Conroy DA, Armitage R, Brower KJ. Cognitive-behavioral therapy for insomnia in alcohol dependent patients: a randomized controlled pilot trial. Behav Res Ther.

2011;49(4):227-33.

12. Yoshimura A, Kimura M, Nakayama H, et al. Efficacy of disulfiram for the treatment of alcohol dependence assessed with a multicenter randomized controlled trial. Alcohol Clin Exp Res. 2014;38(2):572-8.

13. Baker A, Bucci S, Lewin TJ, et al. Cognitive-behavioural therapy for substance use disorders in people with psychotic disorders: Randomised controlled trial. Br J Psychiatry. 2006;188:439-48.

14. Balldin J, Berglund M, Borg S, et al. A 6-month controlled naltrexone study: combined effect with cognitive behavioral therapy in outpatient treatment of alcohol dependence. Alcohol Clin Exp Res. 2003;27(7):1142-9.

15. Baltieri DA, de Andrade AG. Efficacy of acamprosate in the treatment of alcohol-dependent outpatients. Rev Bras Psiquiatr. 2003;25(3):156-9.

16. Baltieri DA, De Andrade AG.Acamprosate in alcohol dependence: a randomized controlled efficacy study in a standard clinical setting. J Stud Alcohol. 2004;65(1):136-9.

17. Baltieri DA, Daró FR, Ribeiro PL, de Andrade AG. Comparing topiramate with naltrexone in the treatment of alcohol dependence. Addiction. 2008;103(12):2035-44.

18. Barnett NP, Celio MA, Tidey JW, et al. A preliminary randomized controlled trial of contingency management for alcohol use reduction using a transdermal alcohol sensor. Addiction. 2017;112(6):1025-1035.

19. Baumann S, Gaertner B, Schnuerer I, et al. The impact of a stage tailored intervention on alcohol use trajectories among those who do not intend to change. Drug Alcohol Depend. 2015;147:167-74.

20. Bender S, Scherbaum N, Soyka M, et al. The efficacy of the dopamine D2/D3 antagonist tiapride in maintaining abstinence: a randomized, double-blind, placebo-controlled trial in 299 alcohol-dependent patients. Int J Neuropsychopharmacol. 2007;10(5):653-60.

21. Bendtsen P, McCambridge J, Bendtsen M, et al. Effectiveness of a proactive mail-based alcohol Internet intervention for university students: dismantling the assessment and feedback components in a randomized controlled trial. J Med Internet Res. 2012;14(5):e142.

22. Berner MM, Wahl S, Brueck R, et al. The place of additional individual psychotherapy in the treatment of alcoholism: a randomized controlled study in nonresponders to anticraving medication-results of the PREDICT study. Alcohol Clin Exp Res. 2014;38(4):1118-25.

23. Blondell RD, Frydrych LM, Jaanimagi U, et al. A randomized trial of two behavioral interventions to improve outcomes following inpatient detoxification for alcohol dependence. J Addict Dis. 2011;30(2):136-48.

24. Carmody TP, Delucchi K, Duncan CL, et al. Intensive intervention for alcohol-dependent smokers in early recovery: a randomized trial. Drug Alcohol Depend. 2012;122(3):186-94.

25. Chang G, Wilkins-Haug L, Berman S, Goetz MA. Brief intervention for alcohol use in pregnancy: a randomized trial. Addiction. 1999;94(10):1499-508.

26. Charney DA, Heath LM, Zikos E, et al. Poorer Drinking Outcomes with Citalopram Treatment for Alcohol Dependence: A Randomized, Double-Blind, Placebo-Controlled Trial. Alcohol Clin Exp Res. 2015;39(9):1756-65.

27. Chick J, Aschauer H, Hornik K; Investigators' Group. Efficacy of fluvoxamine in preventing relapse in alcohol dependence: a one-year, double-blind, placebo controlled multicentre study with analysis by typology. Drug Alcohol Depend. 2004;74(1):61-70.

28. Cornelius JR, Salloum IM, Ehler JG, et al. Fluoxetine in depressed alcoholics. A double-blind, placebo-controlled trial. Arch Gen Psychiatry. 1997;54(8):700-5.

29. Crits-Christoph P, Ring-Kurtz S, McClure B, et al. A randomized controlled study of a web-based performance improvement system for substance abuse treatment providers. J Subst Abuse Treat. 2010;38(3):251-62.

30. Davis WT, Campbell L, Tax J, Lieber CS. A trial of "standard" outpatient alcoholism treatment vs. a minimal treatment control. J Subst Abuse Treat. 2002;23(1):9-19.

31. Dongier M, Vachon L, Schwartz G. Bromocriptine in the treatment of alcohol dependence. Alcohol Clin Exp Res. 1991;15(6):970-7.

32. Drummond C, Gilburt H, Burns T, et al. Assertive Community Treatment For People With Alcohol Dependence: A Pilot Randomized Controlled Trial. Alcohol Alcohol. 2017;52(2):234-241.

33. Elder JP, Litrownik AJ, Slymen DJ, et al. Tobacco and alcohol use-prevention program for Hispanic migrant adolescents. Am J Prev Med. 2002;23(4):269-75.

34. Fawcett J, Clark DC, Aagesen CA, et al. A double-blind, placebo-controlled trial of lithium carbonate therapy for alcoholism. Arch Gen Psychiatry. 1987;44(3):248-56.

35. Fertig JB, Ryan ML, Falk DE, et al. A double-blind, placebo-controlled trial assessing the efficacy of levetiracetam extended-release in very heavy drinking alcohol-dependent patients. Alcohol Clin Exp Res. 2012;36(8):1421-30.

36. Friedmann PD, Rose JS, Swift R, et al. Trazodone for sleep disturbance after alcohol detoxification: a double-blind, placebo-controlled trial. Alcohol Clin Exp Res. 2008;32(9):1652-60.

37. Fuller RK, Roth HP. Disulfiram for the treatment of alcoholism. An evaluation in 128 men. Ann Intern Med. 1979;90(6):901-4.

38. Gallimberti L, Ferri M, Ferrara SD, et al. Gamma-Hydroxybutyric acid in the treatment of alcohol dependence: a double-blind study. Alcohol Clin Exp Res. 1992;16(4):673-6.

39. Garbutt JC, Kampov-Polevoy AB, Gallop R, et al. Efficacy and safety of baclofen for alcohol dependence: a randomized, double-blind, placebo-controlled trial. Alcohol Clin Exp Res. 2010;34(11):1849-57.

40. Geshi M, Hirokawa K, Taniguchi T, et al. Effects of alcohol-related health education on alcohol and drinking behavior awareness among Japanese junior college students: a randomized controlled trial. Acta Med Okayama. 2007;61(6):345-54.

41. Gottlieb LD, Horwitz RI, Kraus ML, et al. Randomized controlled trial in alcohol relapse prevention: role of atenolol, alcohol craving, and treatment adherence. J Subst Abuse Treat. 1994;11(3):253-8.

42. Grønbaek M, Nielsen B. A randomized controlled trial of Minnesota day clinic treatment of alcoholics. Addiction. 2007;102(3):381-8.

43. Gual A, Balcells M, Torres M, et al. Sertraline for the prevention of relapse in detoxicated alcohol dependent patients with a comorbid depressive disorder: a randomized controlled trial. Alcohol Alcohol. 2003;38(6):619-25.

44. Guardia J, Caso C, Arias F, et al. A double-blind, placebo-controlled study of naltrexone in the treatment of alcohol-dependence disorder: results from a multicenter clinical trial. Alcohol Clin Exp Res. 2002;26(9):1381-7.

45. Guardia J, Segura L, Gonzalvo B, et al. A double-blind, placebo-controlled study of olanzapine in the treatment of alcohol-dependence disorder. Alcohol Clin Exp Res. 2004;28(5):736-45.

46. Gustafson DH, McTavish FM, Chih MY, et al. A smartphone application to support recovery from alcoholism: a randomized clinical trial. JAMA Psychiatry. 2014;71(5):566-72.

47. Hagedorn HJ, Noorbaloochi S, Simon AB, et al. Rewarding early abstinence in Veterans Health Administration addiction clinics. J Subst Abuse Treat. 2013;45(1):109-17.

48. Haskins BL, Davis-Martin R, Abar B, et al. Health Evaluation and Referral Assistant: A Randomized Controlled Trial of a Web-Based Screening, Brief Intervention, and Referral to Treatment System to Reduce Risky Alcohol Use Among Emergency Department Patients. J Med Internet Res. 2017;19(5):e119.

49. Hernandez-Avila CA, Modesto-Lowe V, Feinn R, Kranzler HR. Nefazodone treatment of comorbid alcohol dependence and major depression. Alcohol Clin Exp Res. 2004;28(3):433-40.

50. Hien DA, Levin FR, Ruglass LM, et al. Combining seeking safety with sertraline for PTSD and alcohol use disorders: A randomized controlled trial. J Consult Clin Psychol. 2015;83(2):359-69.

51. Johnson BA, Rosenthal N, Capece JA, et al. Topiramate for treating alcohol dependence: a randomized controlled trial. JAMA. 2007;298(14):1641-51.

52. Joos L, Goudriaan AE, Schmaal L, et al. Effect of modafinil on impulsivity and relapse in alcohol dependent patients: a randomized, placebo controlled trial. Eur Neuropsychopharmacol. 2013;23(8):948-55.

53. Kabel DI, Petty F. A placebo-controlled, double-blind study of fluoxetine in severe alcohol dependence: adjunctive pharmacotherapy during and after inpatient treatment. Alcohol Clin Exp Res. 1996;20(4):780-4.

54. Kampman KM, Pettinati HM, Lynch KG, et al. A double-blind, placebo-controlled pilot trial of quetiapine for the treatment of Type A and Type B alcoholism. J Clin Psychopharmacol. 2007;27(4):344-51.

55. Kay-Lambkin FJ, Baker AL, Kelly B, Lewin TJ. Clinician-assisted computerised versus therapist-delivered treatment for depressive and addictive disorders: a randomised controlled trial. Med J Aust. 2011;195(3):S44-50.

56. L'Engle KL, Mwarogo P, Kingola N, et al. A randomized controlled trial of a brief intervention to reduce alcohol use among female sex workers in Mombasa, Kenya. J Acquir Immune Defic Syndr. 2014;67(4):446-53.

57. Yonkers KA, Forray A, Howell HB, et al. Motivational enhancement therapy coupled with cognitive behavioral therapy versus brief advice: a randomized trial for treatment of hazardous substance use in pregnancy and after delivery. Gen Hosp Psychiatry. 2012;34(5):439-49.

58. Kranzler HR, Bauer LO, Hersh D, Klinghoffer V. Carbamazepine treatment of cocaine dependence: a placebo-controlled trial. Drug Alcohol Depend. 1995;38(3):203-11.

59. Kranzler HR, Burleson JA, Korner P, et al. Placebo-controlled trial of fluoxetine as an adjunct to relapse prevention in alcoholics. Am J Psychiatry. 1995;152(3):391-7.

60. Kranzler HR, Modesto-Lowe V, Van Kirk J. Naltrexone vs. nefazodone for treatment of alcohol dependence. A placebo-controlled trial. Neuropsychopharmacology. 2000;22(5):493-503.

61. Kranzler HR, Wesson DR, Billot L, et al. Naltrexone depot for treatment of alcohol dependence: a multicenter, randomized, placebo-controlled clinical trial. Alcohol Clin Exp Res. 2004;28(7):1051-9.

62. LaBrie JW, Huchting KK, Lac A, et al. Preventing risky drinking in first-year college women: further validation of a female-specific motivational enhancement group intervention. J Stud Alcohol Drugs Suppl. 2009;(16):77-85.

63. Latt NC, Jurd S, Houseman J, et al. Naltrexone in alcohol dependence: a randomised controlled trial of effectiveness in a standard clinical setting. Med J Aust. 2002;176(11):530-4.

64. Ledgerwood DM, Petry NM. Does contingency management affect motivation to change substance use? Drug Alcohol Depend. 2006;83(1):65-72.

65. Liddle HA, Dakof GA, Turner RM, et al. Treating adolescent drug abuse: a randomized trial comparing multidimensional family therapy and cognitive behavior therapy. Addiction. 2008;103(10):1660-70.

66. Litt MD, Kadden RM, Cooney NL, Kabela E. Coping skills and treatment outcomes in cognitive-behavioral and interactional group therapy for alcoholism. J Consult Clin Psychol. 2003;71(1):118-28.

67. Litt MD, Kadden RM, Kabela-Cormier E, et al. Changing network support for drinking: initial findings from the network support project. J Consult Clin Psychol. 2007;75(4):542-55.

68. Litten RZ, Fertig JB, Falk DE, et al. A double-blind, placebo-controlled trial to assess the efficacy of quetiapine fumarate XR in very heavy drinking alcohol-dependent patients. Alcohol Clin Exp Res. 2012;36(3):406-16.

69. Litten RZ, Ryan ML, Fertig JB, et al. A double-blind, placebo-controlled trial assessing the efficacy of varenicline tartrate for alcohol dependence. J Addict Med. 2013;7(4):277-86.

70. Lucht MJ, Hoffman L, Haug S, et al. A surveillance tool using mobile phone short message service to reduce alcohol consumption among alcohol-dependent patients. Alcohol Clin Exp Res. 2014;38(6):1728-36.

71. Malcolm R, Anton RF, Randall CL, et al. A placebo-controlled trial of buspirone in anxious inpatient alcoholics. Alcohol Clin Exp Res. 1992;16(6):1007-13.

72. Manning V, Staiger PK, Hall K, et al. Cognitive Bias Modification Training During Inpatient Alcohol Detoxification Reduces Early Relapse: A Randomized Controlled Trial. Alcohol Clin Exp Res. 2016;40(9):2011-9.

73. Marra D, Warot D, Berlin I, et al. Amisulpride does not prevent relapse in primary alcohol dependence: results of a pilot randomized, placebo-controlled trial. Alcohol Clin Exp Res. 2002;26(10):1545-52.

74. Martens MP, Cadigan JM, Rogers RE, Osborn ZH. Personalized drinking feedback intervention for veterans of the wars in Iraq and Afghanistan: a randomized controlled trial. J Stud Alcohol Drugs. 2015;76(3):355-9.

75. Mason BJ, Goodman AM, Chabac S, Lehert P. Effect of oral acamprosate on abstinence in patients with alcohol dependence in a double-blind, placebo controlled trial: the role of patient motivation. J Psychiatr Res. 2006;40(5):383-93.

76. McDonell MG, Srebnik D, Angelo F, et al. Randomized controlled trial of contingency management for stimulant use in community mental health patients with serious mental illness. Am J Psychiatry. 2013;170(1):94-101.

77. McDonell MG, Leickly E, McPherson S, et al. A Randomized Controlled Trial of Ethyl Glucuronide-Based Contingency Management for Outpatients With Co-Occurring Alcohol Use Disorders and Serious Mental Illness. Am J Psychiatry. 2017;174(4):370-377.

78. McGrath PJ, Nunes EV, Stewart JW, et al. Imipramine treatment of alcoholics with primary depression: A placebo-controlled clinical trial. Arch Gen Psychiatry. 1996;53(3):232-40.

79. McKay JR, Lynch KG, Shepard DS, et al. The effectiveness of telephone-based continuing care for alcohol and cocaine dependence: 24-month outcomes. Arch Gen Psychiatry. 2005;62(2):199-207.

80. Miguel AQ, Madruga CS, Cogo-Moreira H, et al. Contingency management is effective in promoting abstinence and retention in treatment among crack cocaine users in Brazil: A randomized controlled trial. Psychol Addict Behav. 2016;30(5):536-543

81. Miller WR, Gribskov CJ, Mortell RL. Effectiveness of a self-control manual for problem drinkers with and without therapist contact. Int J Addict. 1981;16(7):1247-54.

82. Mills KL, Teesson M, Back SE, et al. Integrated exposure-based therapy for co-occurring posttraumatic stress disorder and substance dependence: a randomized controlled trial. JAMA. 2012;308(7):690-9.

83. Moore GF, Williams A, Moore L, Murphy S. An exploratory cluster randomised trial of a university halls of residence based social norms marketing campaign to reduce alcohol consumption among 1st year students. Subst Abuse Treat Prev Policy. 2013;8:15.

84. Morley KC, Teesson M, Reid SC, et al. Naltrexone versus acamprosate in the treatment of alcohol dependence: A multi-centre, randomized, double-blind, placebo-controlled trial. Addiction. 2006;101(10):1451-62.

85. Morley KC, Baillie A, Leung S, et al. Baclofen for the Treatment of Alcohol Dependence and Possible Role of Comorbid Anxiety. Alcohol Alcohol. 2014;49(6):654-60.

86. Morris PL, Hopwood M, Whelan G, et al. Naltrexone for alcohol dependence: a randomized controlled trial. Addiction. 2001;96(11):1565-73.

87. Mueller TI, Stout RL, Rudden S, et al. A double-blind, placebo-controlled pilot study of carbamazepine for the treatment of alcohol dependence. Alcohol Clin Exp Res. 1997;21(1):86-92.

88. Müller CA, Geisel O, Pelz P, et al. High-dose baclofen for the treatment of alcohol dependence (BACLAD study): a randomized, placebo controlled trial. Eur Neuropsychopharmacol. 2015;25(8):1167-77.

89. Mundt JC, Moore HK, Bean P. An interactive voice response program to reduce drinking relapse: a feasibility study. J Subst Abuse Treat. 2006;30(1):21-9.

90. Nadkarni A, Weobong B, Weiss HA, et al. Counselling for Alcohol Problems (CAP), a lay counsellor-delivered brief psychological treatment for harmful drinking in men, in primary care in India: a randomised controlled trial. Lancet. 2017;389(10065):186-195.

91. Nadkarni A, Weiss HA, Weobong B, et al. Sustained effectiveness and cost-effectiveness of Counselling for Alcohol Problems, a brief psychological treatment for harmful drinking in men, delivered by lay counsellors in primary care: 12 month follow-up of a randomised controlled trial. PLoS Med. 2017;14(9):e1002386.

92. Namkoong K, Lee BO, Lee PG, et al. Acamprosate in Korean alcohol-dependent patients: a multi-centre, randomized, double-blind, placebo controlled study. Alcohol Alcohol. 2003;38(2):135-41.

93. Neto D, Lambaz R, Aguiar P, Chick J. Effectiveness of sequential combined treatment in comparison with treatment as usual in preventing relapse in alcohol dependence. Alcohol Alcohol. 2008;43(6):661-8.

94. Niederhofer H, Staffen W, Mair A. Comparison of cyanamide and placebo in the treatment of alcohol dependence of adolescents. Alcohol Alcohol. 2003;38(1):50-3.

95. O'Malley SS, Jaffe AJ, Chang G, et al. Naltrexone and coping skills therapy for alcohol dependence. A controlled study. Arch Gen Psychiatry. 1992;49(11):881-7.

96. O'Malley SS, Sinha R, Grilo CM, et al. Naltrexone and cognitive behavioral coping skills therapy for the treatment of alcohol drinking and eating disorder features in alcohol-dependent women: a randomized controlled trial. Alcohol Clin Exp Res. 2007;31(4):625-34.

97. O'Malley SS, Robin RW, Levenson AL, et al. Naltrexone alone and with sertraline for the treatment of alcohol dependence in Alaska natives and non natives residing in rural settings: a randomized controlled trial. Alcohol Clin Exp Res. 2008;32(7):1271-83.

98. Oslin D, Liberto JG, O'Brien J, et al. Naltrexone as an adjunctive treatment for older patients with alcohol dependence. Am J Geriatr Psychiatry. 1997;5(4):324-32.

99. Oslin DW. Treatment of late-life depression complicated by alcohol dependence. Am J Geriatr Psychiatry. 2005;13(6):491-500.

100. Paille FM, Guelfi JD, Perkins AC, et al. Double-blind randomized multicentre trial of acamprosate in maintaining abstinence from alcohol. Alcohol Alcohol. 1995;30(2):239-47.

101. Pelc I, Verbanck P, Le Bon O, et al. Efficacy and safety of acamprosate in the treatment of detoxified alcohol-dependent patients. A 90-day placebo-controlled dose-finding study. Br J Psychiatry. 1997;171:73-7.

102. Pelc I, Hanak C, Baert I, et al. Effect of community nurse follow-up when treating alcohol dependence with acamprosate. Alcohol Alcohol. 2005;40(4):302-7.

103. Petrakis IL, Poling J, Levinson C, et al. Naltrexone and disulfiram in patients with alcohol dependence and comorbid post-traumatic stress disorder. Biol Psychiatry. 2006;60(7):777-83.

104. Petry NM, Martin B, Cooney JL, Kranzler HR. Give them prizes, and they will come: contingency management for treatment of alcohol dependence. J Consult Clin Psychol. 2000;68(2):250-7.

105. Petry NM, Rash CJ, Easton CJ. Contingency management treatment in substance abusers with and without legal problems. J Am Acad Psychiatry Law. 2011;39(3):370-8.

106. Pettinati HM, Volpicelli JR, Luck G, et al. Double-blind clinical trial of sertraline treatment for alcohol dependence. J Clin Psychopharmacol. 2001;21(2):143-53.

107. Pettinati HM, Kampman KM, Lynch KG, et al. A double blind, placebo-controlled trial that combines disulfiram and naltrexone for treating co-occurring cocaine and alcohol dependence. Addict Behav. 2008;33(5):651-67.

108. Pettinati HM, Oslin DW, Kampman KM, et al. A double-blind, placebo-controlled trial combining sertraline and naltrexone for treating co-occurring depression and alcohol dependence. Am J Psychiatry. 2010;167(6):668-75.

109. Pettinati HM, Kampman KM, Lynch KG, et al. A pilot trial of injectable, extended-release naltrexone for the treatment of co-occurring cocaine and alcohol dependence. Am J Addict. 2014;23(6):591-7.

110. Poldrugo F. Acamprosate treatment in a long-term community-based alcohol rehabilitation programme. Addiction. 1997;92(11):1537-46.

111. Rash CJ, Alessi SM, Petry NM. Cocaine abusers with and without alcohol dependence respond equally well to contingency management treatments. Exp Clin Psychopharmacol. 2008;16(4):275-81.

112. Reback CJ, Peck JA, Dierst-Davies R, et al. Contingency management among homeless, out-of-treatment men who have sex with men. J Subst Abuse Treat. 2010;39(3):255-63.

113. Richter C, Effenberger S, Bschor T, et al. Efficacy and safety of levetiracetam for the prevention of alcohol relapse in recently detoxified alcohol dependent patients: a randomized trial. J Clin Psychopharmacol. 2012;32(4):558-62.

114. Roll JM, Petry NM, Stitzer ML, et al. Contingency management for the treatment of methamphetamine use disorders. Am J Psychiatry. 2006;163(11):1993-9.

115. Saitz R, Palfai TP, Cheng DM, et al. Brief intervention for medical inpatients with unhealthy alcohol use: a randomized, controlled trial. Ann Intern Med. 2007;146(3):167-76.

116. Saitz R, Cheng DM, Winter M, et al. Chronic care management for dependence on alcohol and other drugs: the AHEAD randomized trial. JAMA. 2013;310(11):1156-67.

117. Sass H, Soyka M, Mann K, Zieglgänsberger W. Relapse prevention by acamprosate. Results from a placebo-controlled study on alcohol dependence. Arch Gen Psychiatry. 1996;53(8):673-80.

118. Schumacher JE, Milby JB, Wallace D, et al. Diagnostic compared with abstinence outcomes of day treatment and contingency management among cocaine-dependent homeless persons. Exp Clin Psychopharmacol. 2003;11(2):146-57.

119. Signor L, Pierozan PS, Ferigolo M, et al. Efficacy of the telephone-based Brief Motivational Intervention for alcohol problems in Brazil. Rev Bras Psiquiatr. 2013;35(3):254-61.

120. Sinadinovic K, Wennberg P, Johansson M, Berman AH. Targeting individuals with problematic alcohol use via Web-based cognitive-behavioral self-help modules, personalized screening feedback or assessment only: a randomized controlled trial. Eur Addict Res. 2014;20(6):305-18.

121. Stein MD, Caviness CM, Anderson BJ, et al. A brief alcohol intervention for hazardously drinking incarcerated women. Addiction. 2010;105(3):466-75.

122. O'Malley SS, Rounsaville BJ, Farren C, et al. Initial and maintenance naltrexone treatment for alcohol dependence using primary care vs specialty care: a nested sequence of 3 randomized trials. Arch Intern Med. 2003;163(14):1695-704.

123. Tempesta E, Janiri L, Bignamini A, et al. Acamprosate and relapse prevention in the treatment of alcohol dependence: a placebo-controlled study. Alcohol Alcohol. 2000;35(2):202-9.

124. Tolliver BK, Desantis SM, Brown DG, et al. A randomized, double-blind, placebo-controlled clinical trial of acamprosate in alcohol-dependent individuals with bipolar disorder: a preliminary report. Bipolar Disord. 2012;14(1):54-63.

125. Toneatto T, Brands B, Selby P. A randomized, double-blind, placebo-controlled trial of naltrexone in the treatment of concurrent alcohol use disorder and pathological gambling. Am J Addict. 2009;18(3):219-25.

126. Upshur C, Weinreb L, Bharel M, et al. A randomized control trial of a chronic care intervention for homeless women with alcohol use problems. J Subst Abuse Treat. 2015;51:19-29.

127. Utter GH, Young JB, Theard LA, et al. The effect on problematic drinking behavior of a brief motivational interview shortly after a first arrest for driving under the influence of alcohol: a randomized trial. J Trauma Acute Care Surg. 2014;76(3):661-70; discussion 670-1.

128. Volpicelli JR, Alterman AI, Hayashida M, O'Brien CP. Naltrexone in the treatment of alcohol dependence. Arch Gen Psychiatry. 1992;49(11):876-80.

129. Volpicelli JR, Rhines KC, Rhines JS, et al. Naltrexone and alcohol dependence. Role of subject compliance. Arch Gen Psychiatry. 1997;54(8):737-42.

130. Werch CE, Pappas DM, Carlson JM, DiClemente CC. Short- and long-term effects of a pilot prevention program to reduce alcohol consumption. Subst Use Misuse. 1998;33(11):2303-21.

131. Whitworth AB, Fischer F, Lesch OM, et al. Comparison of acamprosate and placebo in long-term treatment of alcohol dependence. Lancet. 1996;347(9013):1438-42.

132. Wiesbeck GA, Weijers HG, Lesch OM, et al. Flupenthixol decanoate and relapse prevention in alcoholics: results from a placebo-controlled study. Alcohol Alcohol. 2001;36(4):329-34.

133. De Wildt WA, Schippers GM, et al. Does psychosocial treatment enhance the efficacy of acamprosate in patients with alcohol problems? Alcohol Alcohol. 2002;37(4):375-82.

134. Willenbring ML, Olson DH. A randomized trial of integrated outpatient treatment for medically ill alcoholic men. Arch Intern Med. 1999;159(16):1946-52.

135. Winters KC, Fahnhorst T, Botzet A, et al. Brief intervention for drug-abusing adolescents in a school setting: outcomes and mediating factors. J Subst Abuse Treat. 2012;42(3):279-88.

136. Winters KC, Lee S, Botzet A, et al. One-year outcomes and mediators of a brief intervention for drug abusing adolescents. Psychol Addict Behav. 2014;28(2):464-74.

137. Wölwer W, Frommann N, Jänner M, et al. The effects of combined acamprosate and integrative behaviour therapy in the outpatient treatment of alcohol dependence: a randomized controlled trial. Drug Alcohol Depend. 2011;118(2-3):417-22.

Table S3. Sensitivity Analysis: Pooled odds ratios and credible intervals of (*) the main analysis represents our main results and were also presented in Figure 3 in the main text; (a)"USA only" indicates the pooled estimation of data extracted from all USA papers; (b) Data were extracted from treatment and follow-up sessions lasting more than 12 weeks; (c) Trials of reporting discontinuous abstinence were excluded, and all the continuous abstinence data was pooled-estimated in the network meta-analysis. All the results of (a), (b) and (c) were used for comparison with the main analysis (*) to complete sensitivity analysis.

Intervention	Treatment session				Follow-up session			
	Main analysis(*)	USA only(a)	≥ 12 weeks(b)	Continuous abstinent(c)	Main analysis(*)	USA only(a)	≥ 12 weeks(b)	Continuous abstinent(c)
	Compare to BI							
CM	2.75 (0.31, 31.82)	1.23 (0.32, 4.53)	3.23 (0.38, 37.95)	2.70 (0.32, 31.34)	1.47 (0.24, 10.96)	1.85 (0.21, 17.65)	1.36 (0.21, 10.00)	1.53 (0.22, 11.54)
CM + Psychotherapy	4.06 (2.07, 8.18)	2.53 (1.45, 4.44)	3.70 (2.06, 6.51)	5.30 (2.88, 8.05)	0.81 (0.42, 1.52)	0.87 (0.38, 1.87)	0.52 (0.19, 1.45)	0.94 (0.46, 1.89)
Control	0.81 (0.59, 1.12)	0.81 (0.59, 1.09)	0.92 (0.67, 1.26)	0.78 (0.57, 1.07)	0.76 (0.46, 1.22)	0.95 (0.42, 2.04)	0.72 (0.40, 1.25)	0.81 (0.44, 1.46)
Pharmacotherapy	0.96 (0.64, 1.48)	1.08 (0.69, 1.70)	1.32 (0.87, 2.00)	0.92 (0.62, 1.36)	0.52 (0.26, 1.02)	0.49 (0.13, 1.74)	0.53 (0.25, 1.12)	0.50 (0.22, 1.09)
Pharmacotherapy + BI	1.87 (1.40, 2.49)	1.54 (1.08, 2.20)	2.01 (1.54, 2.62)	2.08 (1.65, 2.66)	0.53 (0.39, 0.74)	0.70 (0.33, 1.51)	0.50 (0.34, 0.74)	0.53 (0.38, 0.75)
Pharmacotherapy + Psychotherapy	1.43 (0.96, 2.22)	1.26 (0.87, 1.85)	1.41 (0.98, 2.08)	1.42 (0.98, 2.11)	0.48 (0.27, 0.84)	0.66 (0.27, 1.68)	0.51 (0.25, 1.06)	0.44 (0.21, 0.86)
Psychotherapy	1.23 (0.87, 1.76)	1.10 (0.80, 1.52)	1.14 (0.81, 1.59)	1.35 (0.98, 1.87)	0.75 (0.52, 1.06)	0.84 (0.53, 1.37)	0.80 (0.50, 1.29)	0.72 (0.49, 1.06)
Psychotherapy + BI	1.48 (0.79, 2.78)	1.72 (1.03, 2.95)	1.77 (0.89, 3.47)	1.08 (0.46, 2.52)	1.65 (0.71, 3.92)	1.39 (0.32, 6.03)	1.51 (0.58, 4.22)	1.42 (0.42, 4.85)
	Compare to CM							
CM + Psychotherapy	1.46 (0.12, 14.07)	2.05 (0.52, 8.66)	1.14 (0.10, 10.23)	2.70 (0.19, 27.80)	0.55 (0.07, 3.74)	0.47 (0.05, 4.46)	0.39 (0.04, 3.21)	0.62 (0.07, 4.75)
Control	0.30 (0.03, 2.59)	0.66 (0.18, 2.44)	0.29 (0.03, 2.32)	0.29 (0.03, 2.39)	0.52 (0.07, 3.05)	0.51 (0.06, 4.03)	0.53 (0.08, 3.21)	0.53 (0.07, 3.34)
Pharmacotherapy	0.35 (0.03, 3.13)	0.89 (0.24, 3.41)	0.41 (0.04, 3.44)	0.34 (0.03, 2.80)	0.35 (0.05, 2.18)	0.26 (0.03, 2.58)	0.40 (0.05, 2.65)	0.32 (0.04, 2.28)
Pharmacotherapy + BI	0.68 (0.06, 5.98)	1.25 (0.33, 4.95)	0.62 (0.05, 5.24)	0.77 (0.07, 6.60)	0.36 (0.05, 2.30)	0.38 (0.04, 3.78)	0.37 (0.05, 2.52)	0.35 (0.04, 2.53)
Pharmacotherapy + Psychotherapy	0.51 (0.04, 4.78)	1.03 (0.28, 4.00)	0.44 (0.04, 3.76)	0.53 (0.04, 4.58)	0.33 (0.04, 2.17)	0.36 (0.03, 3.71)	0.38 (0.04, 2.81)	0.28 (0.03, 2.21)
Psychotherapy	0.44 (0.04, 3.99)	0.90 (0.24, 3.48)	0.35 (0.03, 2.95)	0.50 (0.04, 4.31)	0.51 (0.07, 3.19)	0.46 (0.05, 4.09)	0.59 (0.08, 4.09)	0.47 (0.06, 3.30)
Psychotherapy + BI	0.54 (0.04, 5.09)	1.40 (0.35, 5.75)	0.55 (0.04, 5.03)	0.39 (0.03, 4.04)	1.12 (0.14, 8.60)	0.76 (0.05, 10.65)	1.11 (0.12, 9.76)	0.91 (0.09, 9.28)
	Compare to CM + Psychotherapy							
Control	0.20 (0.10, 0.40)	0.32 (0.18, 0.57)	0.25 (0.13, 0.46)	0.11 (0.04, 0.28)	0.93 (0.44, 2.05)	1.09 (0.38, 3.07)	1.38 (0.44, 4.35)	0.87 (0.35, 2.10)
Pharmacotherapy	0.24 (0.11, 0.48)	0.43 (0.22, 0.82)	0.36 (0.19, 0.69)	0.13 (0.05, 0.33)	0.63 (0.26, 1.63)	0.57 (0.13, 2.37)	1.02 (0.31, 3.64)	0.53 (0.19, 1.52)
Pharmacotherapy + BI	0.46 (0.22, 0.95)	0.61 (0.32, 1.17)	0.54 (0.30, 1.02)	0.29 (0.11, 0.74)	0.65 (0.33, 1.35)	0.81 (0.28, 2.57)	0.96 (0.33, 2.77)	0.56 (0.26, 1.22)

Pharmacotherapy + Psychotherapy	0.35 (0.18, 0.68)	0.50 (0.29, 0.86)	0.38 (0.22, 0.67)	0.20 (0.08, 0.48)		0.59 (0.28, 1.29)	0.76 (0.27, 2.24)	0.97 (0.30, 3.19)	0.46 (0.18, 1.15)
Psychotherapy	0.30 (0.17, 0.54)	0.44 (0.27, 0.70)	0.31 (0.19, 0.50)	0.19 (0.08, 0.44)		0.93 (0.51, 1.68)	0.97 (0.48, 2.00)	1.52 (0.58, 4.24)	0.76 (0.39, 1.53)
Psychotherapy + BI	0.37 (0.14, 0.90)	0.69 (0.33, 1.44)	0.48 (0.20, 1.16)	0.15 (0.04, 0.53)		2.04 (0.71, 6.05)	1.60 (0.30, 8.81)	2.87 (0.70, 13.32)	1.52 (0.37, 6.09)
	Compare to Control								
Pharmacotherapy	1.18 (0.88, 1.60)	1.35 (0.91, 1.98)	1.43 (1.05, 1.97)	1.17 (0.87, 1.61)		0.68 (0.40, 1.16)	0.52 (0.18, 1.44)	0.74 (0.41, 1.36)	0.61 (0.32, 1.17)
Pharmacotherapy + BI	2.30 (1.51, 3.51)	1.91 (1.21, 3.03)	2.18 (1.46, 3.27)	2.66 (1.83, 3.93)		0.70 (0.40, 1.26)	0.74 (0.25, 2.30)	0.70 (0.38, 1.35)	0.65 (0.34, 1.27)
Pharmacotherapy + Psychotherapy	1.76 (1.17, 2.73)	1.57 (1.04, 2.32)	1.54 (1.03, 2.36)	1.82 (1.21, 2.78)		0.63 (0.32, 1.31)	0.70 (0.23, 2.20)	0.70 (0.30, 1.72)	0.53 (0.22, 1.29)
Psychotherapy	1.51 (1.04, 2.21)	1.36 (0.95, 1.95)	1.23 (0.85, 1.83)	1.72 (1.18, 2.55)		0.99 (0.57, 1.70)	0.89 (0.41, 1.99)	1.11 (0.58, 2.18)	0.88 (0.45, 1.71)
Psychotherapy + BI	1.82 (0.90, 3.71)	2.14 (1.24, 3.76)	1.91 (0.95, 3.91)	1.37 (0.56, 3.41)		2.18 (0.84, 6.01)	1.47 (0.28, 7.91)	2.09 (0.70, 6.97)	1.74 (0.45, 6.95)
	Compare to Pharmacotherapy								
Pharmacotherapy + BI	1.95 (1.18, 3.18)	1.42 (0.80, 2.49)	1.52 (0.94, 2.48)	2.26 (1.47, 3.50)		1.02 (0.50, 2.14)	1.42 (0.34, 6.85)	0.94 (0.42, 2.10)	1.06 (0.47, 2.45)
Pharmacotherapy + Psychotherapy	1.49 (0.97, 2.34)	1.16 (0.74, 1.87)	1.08 (0.69, 1.69)	1.54 (1.01, 2.41)		0.93 (0.40, 2.21)	1.35 (0.30, 6.47)	0.96 (0.35, 2.60)	0.87 (0.31, 2.39)
Psychotherapy	1.27 (0.84, 1.96)	1.01 (0.65, 1.61)	0.86 (0.55, 1.33)	1.46 (0.98, 2.21)		1.44 (0.70, 3.01)	1.71 (0.48, 6.55)	1.49 (0.65, 3.49)	1.45 (0.61, 3.37)
Psychotherapy + BI	1.54 (0.72, 3.28)	1.59 (0.82, 3.06)	1.34 (0.63, 2.85)	1.17 (0.46, 2.94)		3.20 (1.08, 9.55)	2.85 (0.40, 21.44)	2.81 (0.84, 10.05)	2.88 (0.68, 12.59)
	Compare to Pharmacotherapy + BI								
Pharmacotherapy + Psychotherapy	0.76 (0.47, 1.28)	0.82 (0.49, 1.35)	0.71 (0.46, 1.08)	0.68 (0.45, 1.06)		0.91 (0.50, 1.63)	0.95 (0.29, 3.01)	1.01 (0.48, 2.14)	0.83 (0.38, 1.66)
Psychotherapy	0.66 (0.42, 1.03)	0.71 (0.45, 1.13)	0.57 (0.38, 0.84)	0.65 (0.44, 0.96)		1.41 (0.90, 2.20)	1.21 (0.48, 2.92)	1.59 (0.92, 2.76)	1.36 (0.84, 2.18)
Psychotherapy + BI	0.79 (0.40, 1.58)	1.11 (0.61, 2.08)	0.88 (0.43, 1.84)	0.52 (0.21, 1.25)		3.13 (1.26, 7.74)	1.98 (0.38, 10.36)	2.99 (1.06, 9.13)	2.67 (0.77, 9.60)
	Compare to Pharmacotherapy + Psychotherapy								
Psychotherapy	0.86 (0.63, 1.14)	0.87 (0.65, 1.15)	0.80 (0.61, 1.04)	0.95 (0.71, 1.25)		1.56 (0.94, 2.56)	1.27 (0.58, 2.81)	1.56 (0.85, 2.91)	1.65 (0.89, 3.22)
Psychotherapy + BI	1.03 (0.48, 2.19)	1.36 (0.74, 2.52)	1.25 (0.58, 2.65)	0.75 (0.30, 1.89)		3.47 (1.26, 9.52)	2.11 (0.36, 11.65)	2.96 (0.87, 10.59)	3.29 (0.83, 13.85)
	Compare to Psychotherapy								
Psychotherapy + BI	1.20 (0.58, 2.46)	1.57 (0.87, 2.82)	1.55 (0.75, 3.25)	0.79 (0.32, 2.00)		2.22 (0.89, 5.63)	1.67 (0.35, 7.70)	1.90 (0.65, 6.00)	1.98 (0.56, 7.14)

Table S4. Pairwise meta-analysis of different interventions versus controls regarding treatment sessions and follow-up sessions. The effects of overall and each intervention regarding treatment sessions and follow-up sessions were presented. [OR: odds ratio; CI: credible intervals].

Intervention		No. of reports	Data Synthesis		
			**OR (95% CI)**	**I-squared**	**P value**
**Treatment session**	Overall				
	Intervention vs. Control	60	1.260 (1.091, 1.455)	67.30%	0.002
	Subgroup				
	Pharmacotherapy + Psychotherapy vs. Control	2	1.178 (1.002, 1.386)	15.30%	0.048
	Psychotherapy vs. Control	5	1.052 (0.907, 1.220)	22.70%	0.501
	Pharmacotherapy vs. Control	27	1.074 (1.002, 1.152)	47.70%	0.045
	CM vs. Control	2	1.306 (1.048, 1.627)	81.90%	0.017
	BI vs. Control	22	1.064 (1.003, 1.128)	78.60%	0.038
	Psychotherapy + BI vs. Control	2	1.500 (1.055, 2.133)	29.00%	0.024
**Follow-up session**	Overall				
	Intervention vs. Control	16	1.252 (1.012, 1.550)	12.20%	0.039
	Subgroup				
	BI vs. Control	5	1.062 (0.928, 1.216)	43.50%	0.381
	Pharmacotherapy + BI vs. Control	1	—	—	—
	Pharmacotherapy vs. Control	6	1.442 (1.094, 1.900)	0.00%	0.009
	Psychotherapy vs. Control	3	0.967 (0.552, 1.693)	0.00%	0.906
	CM vs. Control	1	—	—	—

Table S5. Pairwise meta-analysis of best rank intervention versus other interventions. Data of best rank intervention of treatment session (CM + psychotherapy) and follow-up session (pharmacotherapy + psychotherapy) were pooled-estimated. The effects of overall and each intervention were presented [OR: odds ratios; CI: credible intervals].

	**Intervention**	**No. of reports**	**Data Synthesis**		
			**OR (95% CI)**	**I-squared**	**P value**
**Treatment session**	Overall				
	CM + Psychotherapy vs. Other Interventions	11	2.191 (1.290, 3.720)	70.70%	0.004
	Subgroup				
	CM + Psychotherapy vs. BI	1	—	—	—
	CM + Psychotherapy vs. Psychotherapy	10	2.277 (1.271, 4.081)	73.60%	0.006
**Follow-up session**	Overall				
	Pharmacotherapy + Psychotherapy vs. Other Interventions	9	1.409 (1.079, 1.840)	0.00%	0.012
	Subgroup				
	Pharmacotherapy + Psychotherapy vs. Pharmacotherapy	1	—	—	—
	Pharmacotherapy + Psychotherapy vs. Pharmacotherapy + BI	2	1.302 (0.830, 2.041)	0.00%	0.251
	Pharmacotherapy + Psychotherapy vs. Psychotherapy	6	1.410 (1.005, 1.978)	0.00%	0.046

Table S6. A node-splitting model was used to test the direct and indirect effects. Potential inconsistency may exist if P-value < 0.05 and indicates results' lack of robustness.

Treatment session				
**Comparison**	**Direct Effect**	**Indirect Effect**	**Overall**	**P-Value**
BI vs. CM + Psychotherapy	0.42 (-0.95, 1.78)	1.77 (1.02, 2.56)	1.39 (0.74, 2.08)	0.07
BI vs. Control	-0.36 (-0.72, 0.00)	0.27 (-0.36, 0.91)	-0.20 (-0.52, 0.11)	0.07
BI vs. Pharmacotherapy + BI	0.64 (0.34, 0.93)	6.92E62 (-4.86E62, 7.86E62)	0.63 (0.34, 0.91)	0.86
BI vs. Pharmacotherapy + Psychotherapy	1.26 (-0.16, 2.64)	0.26 (-0.15, 0.70)	0.36 (-0.05, 0.79)	0.17
BI vs. Psychotherapy	0.39 (-0.03, 0.81)	-0.12 (-0.67, 0.43)	0.20 (-0.14, 0.56)	0.13
BI vs. Psychotherapy + BI	0.38 (-0.23, 1.00)	1.79E62 (-2.55E62, 7.78E62)	0.40 (-0.22, 1.01)	0.68
Control vs. Pharmacotherapy	0.19 (-0.11, 0.49)	-0.24 (-1.37, 0.03)	0.16 (-0.13, 0.47)	0.13
Control vs. Pharmacotherapy + Psychotherapy	0.14 (-0.62, 0.93)	0.72 (0.26, 1.22)	0.56 (0.15, 1.00)	0.21
Control vs. Psychotherapy	-0.10 (-0.76, 0.56)	0.60 (0.16, 1.04)	0.40 (0.04, 0.78)	0.08
Control vs. Psychotherapy + BI	1.13E62 (-3.33E62, 1.31E62)	0.59 (-0.10, 1.31)	0.60 (-0.09, 1.28)	0.62
Pharmacotherapy vs. Pharmacotherapy + BI	-4.48E62 (-1.09E62, 8.7E62)	0.67 (0.18, 1.16)	0.66 (0.16, 1.15)	0.61
Pharmacotherapy vs. Pharmacotherapy + Psychotherapy	0.41 (-0.21, 1.07)	0.30 (-0.28, 0.89)	0.39 (-0.04, 0.85)	0.80

Follow-up session				
**Comparison**	**Direct Effect**	**Indirect Effect**	**Overall**	**P-Value**
BI vs. CM + Psychotherapy	0.39 (-0.47, 1.27)	0.11 (-0.88, 1.12)	0.21 (-0.42, 0.86)	0.66
BI vs. Control	0.03 (-0.52, 0.61)	0.77 (-0.06, 1.61)	0.27 (-0.20, 0.78)	0.15
BI vs. Pharmacotherapy	0.67 (-0.71, 2.11)	0.65 (-0.11, 1.44)	0.66 (-0.02, 1.34)	0.98
BI vs. Pharmacotherapy + BI	0.71 (0.37, 1.05)	0.20 (-0.77, 1.13)	0.63 (0.30, 0.95)	0.29
BI vs. Psychotherapy	0.30 (-0.13, 0.72)	0.31 (-0.33, 0.92)	0.29 (-0.06, 0.65)	0.99
Control vs. Pharmacotherapy + BI	-0.29 (-1.71, 1.08)	0.46 (-0.16, 1.05)	0.36 (-0.23, 0.91)	0.31
Control vs. Psychotherapy	-0.64 (-1.63, 0.33)	0.30 (-0.35, 0.92)	0.01 (-0.53, 0.56)	0.12
Pharmacotherapy vs. Pharmacotherapy + BI	-0.53 (-1.94, 0.88)	0.10 (-0.68, 0.85)	-0.02 (-0.76, 0.69)	0.41
Pharmacotherapy + BI vs. Pharmacotherapy + Psychotherapy	0.29 (-0.58, 1.14)	-0.01 (-0.81, 0.77)	0.10 (-0.49, 0.69)	0.61
Pharmacotherapy + BI vs. Psychotherapy	-0.02 (-1.15, 1.12)	-0.40 (-0.87, 0.08)	-0.34 (-0.79, 0.11)	0.52
Pharmacotherapy + Psychotherapy vs. Psychotherapy	-0.33 (-0.87, 0.23)	-0.80 (-1.80, 0.17)	-0.45 (-0.94, 0.06)	0.37

Pharmacotherapy vs. Psychotherapy	-0.18 (-0.96, 0.61)	0.31 (-0.16, 0.77)	0.24 (-0.19, 0.67)	0.29		
Pharmacotherapy + BI vs. Pharmacotherapy + Psychotherapy	0.66 (-0.65, 2.05)	-0.39 (-0.89, 0.13)	-0.27 (-0.76, 0.25)	0.14		
Pharmacotherapy + BI vs. Psychotherapy	-0.42 (-1.97, 1.04)	-0.43 (-0.87, 0.03)	-0.42 (-0.86, 0.03)	0.99		
Pharmacotherapy + BI vs. Psychotherapy + BI	3.56E62 (-1.64E62, 6.73E62)	-0.24 (-0.91, 0.46)	-0.23 (-0.90, 0.45)	0.33		
Pharmacotherapy + Psychotherapy vs. Psychotherapy	-0.10 (-0.40, 0.20)	-0.28 (-1.04, 0.46)	-0.16 (-0.46, 0.14)	0.65		
Pharmacotherapy + Psychotherapy vs. Psychotherapy + BI	5.55E62 (-6.13E62, 1.22E62)	0.01 (-0.74, 0.77)	0.04 (-0.72, 0.75)	0.99		

Table S7. Overall GRADE quality of evidence. The quality was rated by direct comparison and indirect estimation. Higher ratings were assigned to network meta-analysis estimates if the ratings were coherent between direct and indirect comparisons.

Outcomes	Comparison		Quality of the evidence (Treatment Session)	Quality of the evidence (Follow-up Session)
Abstinent Rate	BI	CM	⊕	⊕
		CM + Psychotherapy	⊕	⊕
		Control	⊕ ⊕ ⊕ ⊕	⊕ ⊕ ⊕ ⊕
		Pharmacotherapy	⊕ ⊕	⊕ ⊕
		Pharmacotherapy + BI	⊕ ⊕ ⊕ ⊕	⊕ ⊕ ⊕
		Pharmacotherapy + Psychotherapy	⊕ ⊕ ⊕	⊕ ⊕
		Psychotherapy	⊕ ⊕ ⊕	⊕ ⊕ ⊕
		Psychotherapy + BI	⊕ ⊕	⊕ ⊕
	CM	CM + Psychotherapy	⊕	⊕
		Control	⊕	⊕
		Pharmacotherapy	⊕ ⊕	⊕ ⊕
		Pharmacotherapy + BI	⊕ ⊕	⊕
		Pharmacotherapy + Psychotherapy	⊕ ⊕	⊕ ⊕
		Psychotherapy	⊕ ⊕	⊕ ⊕
		Psychotherapy + BI	⊕ ⊕	⊕ ⊕
	CM + Psychotherapy	Control	⊕ ⊕	⊕
		Pharmacotherapy	⊕	⊕
		Pharmacotherapy + BI	⊕ ⊕	⊕
		Pharmacotherapy + Psychotherapy	⊕ ⊕ ⊕	⊕ ⊕
		Psychotherapy	⊕ ⊕ ⊕	⊕ ⊕ ⊕
		Psychotherapy + BI	⊕ ⊕ ⊕	⊕ ⊕
	Control	Pharmacotherapy	⊕ ⊕ ⊕ ⊕	⊕ ⊕ ⊕
		Pharmacotherapy + BI	⊕ ⊕ ⊕	⊕

		Pharmacotherapy + Psychotherapy	⊕⊕⊕	⊕⊕⊕
		Psychotherapy	⊕⊕	⊕
		Psychotherapy + BI	⊕⊕⊕	⊕⊕
	Pharmacotherapy	Pharmacotherapy + BI	⊕⊕⊕	⊕⊕
		Pharmacotherapy + Psychotherapy	⊕⊕⊕	⊕⊕
		Psychotherapy	⊕	⊕⊕
		Psychotherapy + BI	⊕⊕⊕	⊕⊕
	Pharmacotherapy + BI	Pharmacotherapy + Psychotherapy	⊕⊕⊕	⊕⊕
		Psychotherapy	⊕⊕⊕	⊕⊕
		Psychotherapy + BI	⊕⊕⊕	⊕⊕
	Pharmacotherapy + Psychotherapy	Psychotherapy	⊕⊕⊕⊕	⊕⊕⊕
		Psychotherapy + BI	⊕⊕⊕	⊕⊕
	Psychotherapy	Psychotherapy + BI	⊕	⊕

GRADE Working Group grades of evidence
High quality (⊕⊕⊕⊕): Further research is very unlikely to change our confidence in the estimate of effect.
Moderate quality (⊕⊕⊕): Further research is likely to have an important impact on our confidence in the estimate of effect and may change the estimate.
Low quality (⊕⊕): Further research is very likely to have an important impact on our confidence in the estimate of effect and is likely to change the estimate.
Very low quality (⊕): We are very uncertain about the estimate.
